# Advanced Multifunctional Optical Coatings for Transparent Glazing: Materials Chemistry, Microstructure, Structure–Property Relationships, and Greenhouse Applications—A Review

**DOI:** 10.3390/ijms27177750

**Published:** 2026-08-29

**Authors:** L. Vijayalakshmi, K. Naveen Kumar, Kishor Palle, Jiseok Lim

**Affiliations:** 1 School of Mechanical Engineering, Yeungnam University, Gyeongsan-si 38541, Republic of Korea; 2 Department of H&S (Chemistry), CVR College of Engineering, Hyderabad 501510, Telangana, India

**Keywords:** functional thin-film coatings, optical coatings, self-cleaning and anti-fog surfaces, transparent substrates (glass and polymers), greenhouse glazing, spectral selectivity, nanostructured oxide coatings

## Abstract

Transparent glazing systems are increasingly required to provide simultaneous control over light transmission, solar heat gain, thermal losses, surface contamination, and environmental durability, creating new challenges for the development of multifunctional coating technologies. This review critically examines advanced optical and self-cleaning coatings developed for transparent glass and polymeric substrates, with particular emphasis on the relationships between materials chemistry, surface/interface chemistry, microstructure, and functional performance. Dielectric multilayers, metal oxides, ceramic coatings, sol-gel-derived hybrid systems, and emerging chromogenic materials are discussed in terms of their chemical compositions, structural characteristics, and mechanisms governing optical, thermal, and surface properties. Particular attention is given to structure–property relationships associated with photosynthetically active radiation (PAR) transmission, near-infrared (NIR) management, thermal emissivity, solar modulation, wettability, and self-cleaning behavior, together with their implications for energy-efficient transparent glazing and greenhouse environments. The influence of coating architecture, porosity, surface roughness, interfacial interactions, and deposition conditions on functional performance and long-term stability is critically evaluated. The advantages and limitations of representative deposition strategies are further compared, considering scalability, process compatibility, substrate sensitivity, and application to heat-sensitive polymeric films. Environmental degradation mechanisms induced by ultraviolet irradiation, moisture, thermal cycling, and mechanical stresses are analyzed to identify the key factors governing coating durability and sustainability. Finally, current knowledge gaps and emerging research directions are identified, highlighting the need for rational materials design, multifunctional integration, scalable fabrication, and improved structure-property-durability correlations for next-generation transparent glazing and greenhouse applications.

## 1. Introduction

Transparent greenhouse glazing is no longer regarded solely as a passive construction material; it can also serve as a functional platform for thin films and surface treatments that modify its optical, thermal, and interfacial properties [1,2,3]. The use of functional coatings allows for selective tuning of the solar spectra, wetting properties of the surface, and improves the material resistance, all essential for providing consistent microclimatic conditions and increasing the operational lifespan of constructions under harsh outdoor conditions [1,4]. In particular, optical and self-cleaning coatings have proven their importance by allowing for simultaneous increase in PAR transmittance while reducing near-infrared heat and UV-induced fading, as well as decreasing soiling-induced loss in transmittance [5,6,7,8].

From a material science perspective, greenhouse glazing represents a challenging case study for transparent coatings in terms of intensive solar radiation, wide variations in temperature and humidity, mechanical stress due to wind or hail pressure, and permanent exposure to dust particles, organic contamination, and agricultural chemicals [2,9]. Therefore, there is a clear necessity to develop strong inorganic and hybrid coating systems based on dielectric layers for spectral control of solar radiation, nanocrystalline metal oxides and ceramics for photocatalytic self-cleaning, and thermally and photochromically responsive layers for optical property adaptation according to environmental conditions [5,6,10,11,12]. This requires careful tailoring of coating composition, microstructure, porosity, and surface chemistry for high transparency to visible light and photosynthetically active radiation while providing the required reflectivity in the near-infrared, UV-blocking capability, wetting properties, and resistance to wear and tear [4,7,11,13].

The recent development of solar control and self-cleaning windows for building and greenhouse applications has shown that appropriate coating formulations can greatly decrease energy consumption requirements due to heating and air conditioning, improve the spectral selectivity performance compared to bare substrates, and increase transparency owing to reduced soiling and a rain-induced cleaning effect [3,5,7,14,15,16]. Such improvements are enabled by many coating processes used, such as physical and chemical vapor deposition, magnetron sputtering, sol–gel technique, and solution-based coating, allowing for the control of thin-film thickness, crystallinity, defect concentration, and coating–substrate interaction strength [1,4,13]. Meanwhile, the issue of stability during prolonged UV exposure, humid conditions, thermal treatment, and mechanical stress, leading to photocatalyst deactivation, alteration of wettability, cracking, and peeling off, is still crucial for the field [2,8,11,15].

Unlike conventional architectural glazing, greenhouse glazing systems must satisfy highly specialized optical and environmental requirements because the transmitted solar spectrum directly influences plant physiology, crop productivity, and greenhouse energy demand. In greenhouse applications, coatings should maintain a high transmission of photosynthetically active radiation (PAR, 400–700 nm), which is essential for photosynthesis and biomass accumulation, while selectively suppressing excessive near-infrared (NIR, 700–2500 nm) radiation responsible for overheating and increased cooling requirements. At the same time, proper management of ultraviolet (UV) radiation is necessary because UV-A (315–400 nm) can contribute positively to plant morphology, pigmentation, and disease resistance, whereas excessive UV-B (280–315 nm) may damage plant tissues and accelerate degradation of polymeric greenhouse materials. Therefore, coating systems developed for greenhouse glazing require a careful balance between spectral selectivity, thermal management, and long-term durability under agricultural operating conditions, including persistent humidity, fertilizer residues, agrochemical exposure, dust accumulation, and repetitive thermal cycling.

The current review presents a materials-centric discussion of modern optical and self-cleaning coatings for transparent substrates in greenhouse glazing applications. On the basis of new trends in the field of functional thin-film and nanocoatings, we firstly identify major coating material systems and deposition processes used for glass and plastic glazing, considering the relations between composition, microstructure, and properties [1,2,3,4,10]. Then, we consider critical performance criteria for greenhouse applications, such as PAR region transparency, infrared transmittance, thermal emissivity, surface energy, self-cleaning properties, and mechanical strength, along with conventional characterization techniques [5,6,7,8,13]. Particular attention is paid to environmental stability and failure mechanisms in real-world greenhouse glazing before outlining future prospects for novel multifunctional adaptive coatings for next-generation greenhouse glazing and transparent envelope systems [3,5,14,16].

Although previous reviews have extensively examined self-cleaning glazing, low-emissivity materials, smart windows, and solar-control coatings, these studies have mainly focused on individual functionalities, material classes, or architectural applications. The present review adopts an integrated materials-science approach by connecting materials chemistry and processing with microstructure, structure–property relationships, durability, and greenhouse-level performance. Particular emphasis is placed on how coating composition, microstructure, interfaces, and processing conditions influence optical, thermal, surface, and mechanical properties and, ultimately, the suitability of coatings for greenhouse environments. This framework considers the simultaneous requirements of high PAR transmission, NIR/UV management, thermal regulation, self-cleaning, and long-term environmental stability, thereby providing a material-processing-structure-property-durability perspective for multifunctional greenhouse glazing coatings.

## 2. Classes of Coating Materials for Transparent Glazing

The materials that act as the basis of greenhouse glazing are soda-lime glass, polycarbonate (PC), polymethyl methacrylate (PMMA), and polyethylene (PE) films. These materials serve as the material substrates on which advanced functional coating structures can be built [1,2,9,17]. The mechanical strength, transmission, and inherent heat insulation provided by the aforementioned substrate materials set the parameters for developing optical coating layers and self-cleaning coatings since processing conditions are significantly constrained by the material’s properties [9,17,18]. Glass possesses good transparency and resistance to chemical attack; however, it lacks thermal insulation and resistance to impacts, while plastic films have lower density and good resistance to impact.

To material scientists, these substrates may be considered as bases for coating architecture design that would provide selective spectral properties, adjustable surface wettability, and increased environmental stability. High-temperature stable dielectric structures, metallic and metal–dielectric multilayer stacks, and dense oxide layers may be applied to glass via sputtering or CVD techniques to ensure precise optical characteristics and high barrier capability [4,16,19,20]. Low-temperature sol–gel, ultraviolet light-curing, or solution-based hybrid coating materials are recommended for thermoplastics like PC and PMMA to prevent thermal/mechanical degradation and delamination due to stress [10,21,22]. For the flexible polyethylene substrates, which are extensively utilized as greenhouse covering membranes, the functional layers should exhibit good adhesion under substantial mechanical bending and weathering conditions while maintaining a high level of PAR transmission [18,21]. A detailed comparison of substrate compatibility and coating functions is provided in Table 1.

### 2.1. Soda-Lime Glass as a Substrate for Functional Coatings

Soda-lime silicate glass continues to be the most widely used glass substrate due to its high visible light transmission rate, chemical stability, and durable nature (lasting over 20 years in some applications) [9,17]. Clear uncoated glass is able to transmit up to 80–90% of incident visible light rays, including the photosynthetic active radiation region, but does not provide good thermal insulation and UV or NIR radiation regulation [1,3,17]. Coatings that can be applied on glass substrates include:

- Multilayer films consisting of dielectrics and dielectric–metal layers that are used for solar and emissivity control [4,16,23].
- UV-blocking and photocatalytic oxide films based on TiO_2_, ZnO, and related systems [21,24].
- Superhydrophobic and hydrophilic silica-based nanofilms that exhibit self-cleaning properties and anti-fogging [7,28].

**Figure 1:**
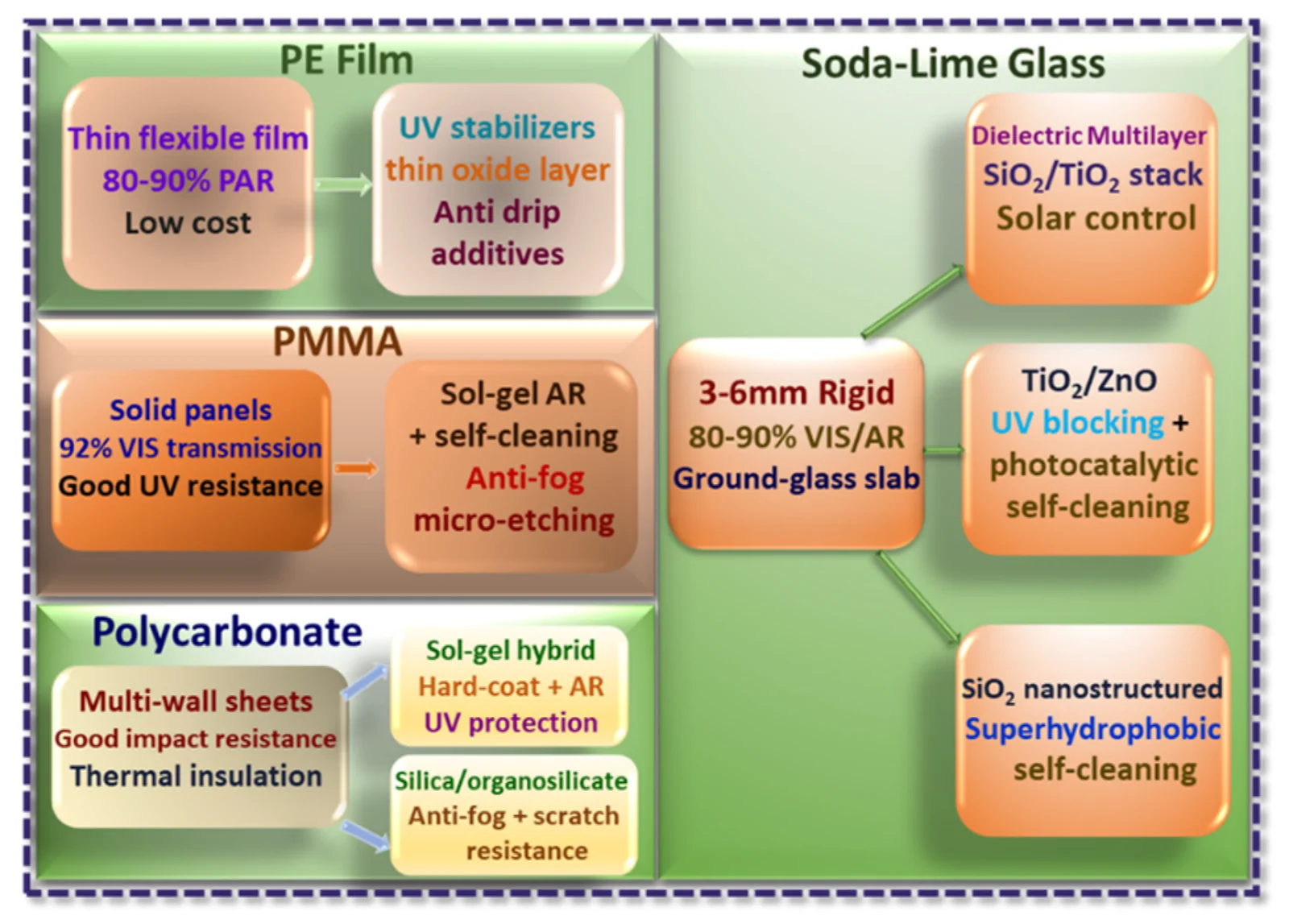
Schematic of substrates and coating architectures for greenhouse glazing.

Due to its ability to withstand high temperatures during fabrication, it allows the formation of sputtered and CVD coatings along with thermally annealed coatings that exhibit controlled crystallinity and density; such characteristics make them suitable for use in greenhouses due to their long-term stability [4,19,20]. The diversity in combination of substrates and coatings for use in glazing for greenhouses is depicted in Figure 1.

### 2.2. Polycarbonate and PMMA Sheets

Both polycarbonate and PMMA plates find wide applications as rigid or semirigid greenhouses because of low density, high impact strength (especially PC), and better thermal insulating properties [18,25]. However, due to low surface hardness, high coefficient of thermal expansion, and tendency to yellowing as a result of UV exposure, coating becomes a necessity [1,18,25]. Hardening or UV-protective coatings are commonly applied to PC in order to improve scratch resistance and prevent photodegradation, usually using the sol–gel process with nanomaterials based on organosilicate or silica chemistry [6,22,26,27]. For the PMMA plate, which already exhibits outstanding optical and UV-resistance properties, anti-reflection, anti-fogging, or hydrophilic self-cleaning coatings can be designed without compromising its transparency much [22,27].

The development of coating for PC and PMMA should take into account that the thermal and mechanical properties of the coating should be properly matched with those of the substrate; besides, the process temperature should not exceed the range of up to 120–140 °C [10,22,27]. Solution-coated and UV-curable coatings appear to be particularly promising in this regard.

### 2.3. Polyethylene Films and Flexible Glazing Substrates

Low-density and linear-low-density polyethylene (LDPE/LLDPE) films are widely employed in greenhouses as flexible covers because of their relatively low prices and good light transmission properties (up to 80–90% for PAR radiation). However, their relatively short lifetime (3–5 years), UV sensitivity, and the fact that these materials become easily contaminated with dust and condensed water make it necessary to use certain additives and thin layers [17,18,21].

It is common practice that commercial PE films contain UV absorbers, IR-absorbing or reflective additives, and wetting agents to impart anti-drip and anti-fog capabilities [18,21]. From a coating perspective, attention has focused on the deposition of oxides or mixed oxide-based thin films at low temperatures that can provide UV shielding, spectral selectivity, and wetting while not compromising mechanical integrity [21,24,27]. The extreme demands posed by the thermal and mechanical properties of the PE substrate have made it an ideal yet difficult material on which to test coating technologies applicable to greenhouses.

### 2.4. Smart and Nanostructured Coating Architectures on Transparent Substrates

In addition, smart coatings and nanoengineered coatings can be used to provide further freedom for customizing the greenhouse microclimate [15,16,29,30]. Thermochromic VO_2_ films deposited on glass permit dynamic control of the transmission of solar radiation and infrared radiation by taking into account changes in temperature [29,30,31]. This approach provides a solution to the problem of energy conservation under conditions of high irradiation intensity. The use of metallic–dielectric thin-film coatings permits an optimization of luminous and thermal properties that depends on local weather conditions. In this regard, numerous simulations and applications in construction for glazing windows were described in the literature [16,23,32]. This approach can be readily used for designing the roofs and sidewalls of greenhouses, since energy savings and microclimate stability play a crucial role there as well [1,2,29].

**Figure 2:**
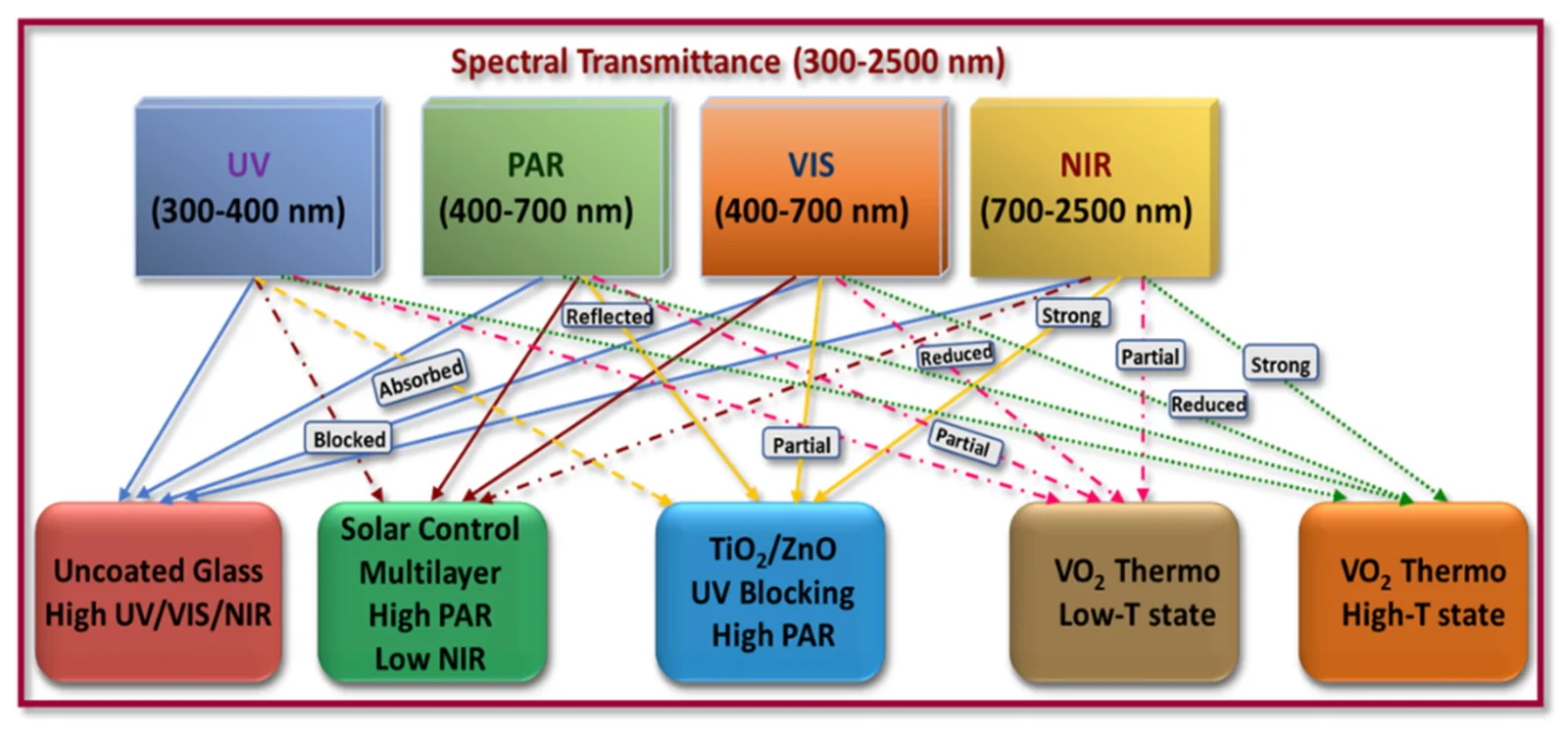
Schematic representation of spectral regions and coating functions for greenhouse glazing.

Superhydrophobic or superhydrophilic nanostructured coatings, usually prepared using silica or titania nanoparticles through sol–gel and hydrothermal processes, have shown the feasibility of obtaining water contact angles higher than 150° or close to 0° while maintaining high levels of transparency [7,28,33,34]. Such coating layers could greatly minimize soiling-induced energy loss and improve light transmission performance by facilitating fast droplet removal (superhydrophobic) or allowing continuous shedding and photocatalytic removal of contaminants (superhydrophilic) [7,24,28]. This is an important way to incorporate such coating architectures onto the substrate materials mentioned previously in order to produce advanced glazing structures for greenhouses. Some representative examples of spectral transmittance characteristics in Figure 2 show their ability to block UV, maintain PAR, and reflect NIR according to greenhouse application requirements.

From a materials-chemistry perspective, the functionality of these coatings is governed by the chemical bonding, composition, phase stability, defect chemistry, and surface reactions of the constituent materials. Wide-band-gap oxides such as TiO_2_, ZnO, and SiO_2_ derive their optical and surface functions from their electronic structures and metal–oxygen bonding environments, while their phase composition, crystallinity, oxygen-vacancy concentration, and surface hydroxyl groups can strongly influence photocatalytic activity, wettability, and spectral response. In metal–dielectric low-emissivity stacks, the chemical stability and oxidation resistance of the metallic layer, together with the refractive index and electronic properties of the dielectric layers, determine infrared reflection, visible transmission, and interfacial stability. For thermochromic oxides such as VO_2_, the reversible semiconductor–metal transition is closely associated with changes in electronic structure and V–O bonding, while dopants and defects can modify the transition temperature and optical modulation. Similarly, in sol-gel-derived hybrid coatings, precursor chemistry, hydrolysis and condensation reactions, cross-linking, and organic–inorganic interactions determine network formation, porosity, surface functionality, and mechanical stability. Thus, chemical composition and bonding provide the fundamental basis from which microstructure develops and ultimately controls the functional performance of greenhouse glazing coatings.

## 3. Deposition and Processing Techniques

The characteristics of both optical and self-cleaning coatings on transparent substrates depend not only on their compositions but also on the coating method, which determines the uniformity of film thickness, the structure of the films, defect concentration, and the degree of adhesion [35,36]. In order to produce coatings on glazing materials for greenhouses, it is necessary that the selected technique will be able to produce uniform coatings over large areas on both glass and polymers at an industrially acceptable rate of production and be compatible with restrictions on substrate temperatures and costs [37,38]. Importantly, deposition conditions also influence the chemical state of the growing film, including stoichiometry, oxidation state, defect concentration, precursor conversion, and surface functional groups, thereby linking processing conditions to subsequent microstructural development and functional properties. The following section presents a summary of main vacuum deposition, chemical, and liquid phase approaches used to produce functional coatings for glazing applications and describes the importance of post-treatments for achieving a desired microstructure and performance [35,36,37,38,39].

### 3.1. Vacuum-Based Deposition (PVD, Sputtering, Evaporation)

Physical vapor deposition (PVD) methods, like thermal evaporation and magnetron sputtering, are widely used for the growth of dense oxide, nitride, and metal–dielectric nanolaminate films on glass supports [35,36]. Thermal evaporation involves heating the coating material to its vapor phase in a high vacuum environment, which then deposits onto the cold substrate; this method facilitates fast coating deposition of simple layered structures and metals [35]. On the other hand, magnetron sputtering uses plasma bombardment to eject atoms from a solid-state target; it enables accurate manipulation of the composition, thickness, and structure of oxide and oxynitride layers, such as TiO_2_, SiO_2_, SnO_2_, and ITO [36,40].

**Figure 3:**
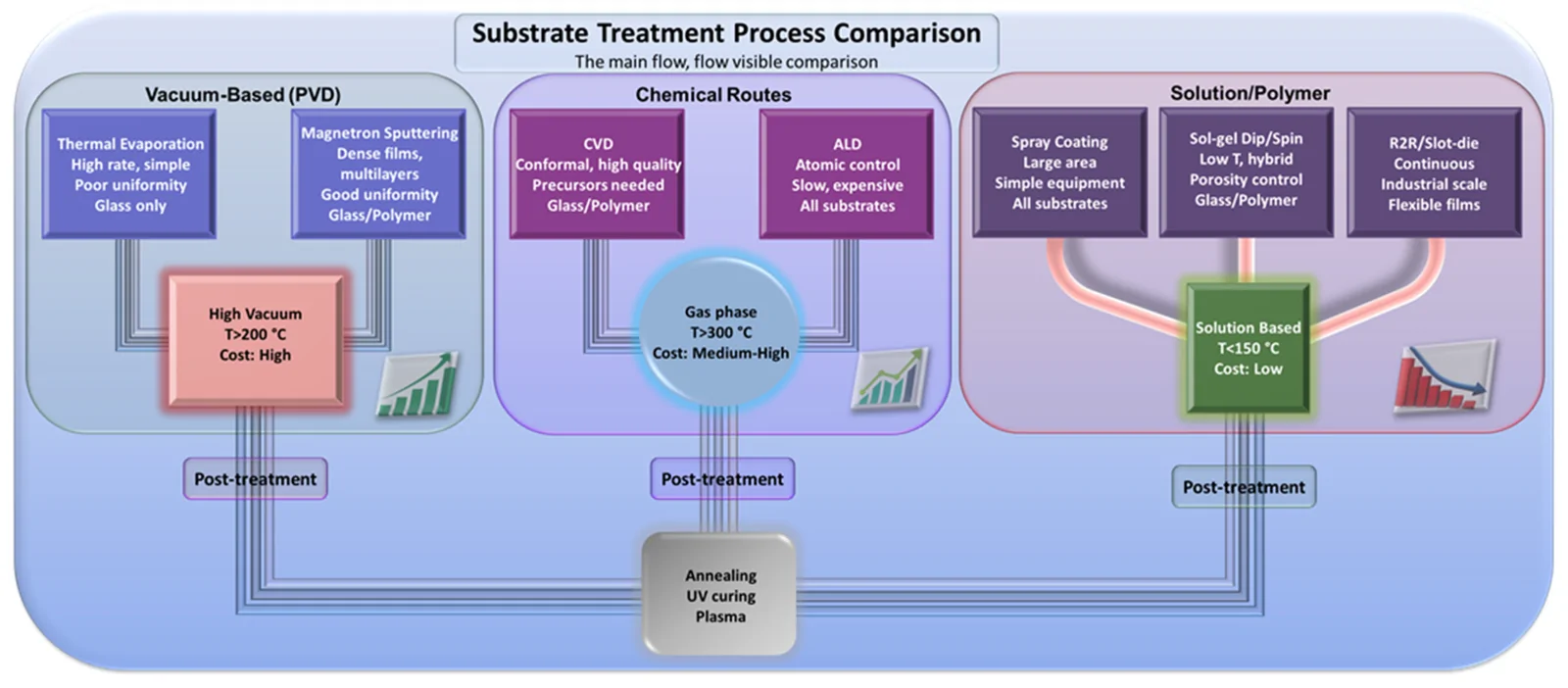
Comparison of deposition techniques for functional coatings.

Reactive sputtering under conditions of oxygen or nitrogen atmosphere becomes especially favorable in cases of solar-control multilayers and emissivity-reducing films, which require precise deposition of high- and low-index layers at a nanometer scale [40,41]. The relatively low substrate temperature achievable in current state-of-the-art sputtering equipment further allows for coating of sensitive-to-heat polymer substrates when appropriate interlayers and measures to reduce stresses are used [40,42]. Also, multi-cathode sputtering technology makes it possible to deposit a complex stack, such as a metal-dielectric-metal or oxide-VO_2_-oxide structure, without exposure to air [40,41,42]. Figure 3 shows the categorization of deposition processes, indicating superiority of vacuum processes in glass multilayer coatings and dominance of solution processes in polymer coatings.

### 3.2. Chemical Routes (CVD, ALD, Sol–Gel Dip/Spin/Spray Coating)

Chemical vapor deposition (CVD) and atomic layer deposition (ALD) are complementary techniques for depositing conformal films with controlled chemistry on transparent substrates [37,43]. During the process of CVD, the volatile precursors undergo reaction or decomposition on the heated substrate surface, leading to the formation of solid films [43]. Oxides, nitrides, and oxynitrides of high quality can be deposited using this technique with superior step coverage and adherence properties. Atmospheric and low-pressure CVD are two reliable methods employed extensively in the glass industry for coating pyrolytic materials like tin oxide and FTO (fluorine-doped tin oxide) onto float glass [37,43].

ALD, based on sequential, self-limiting surface reactions, allows for near-atomic-level control of layer thickness and composition, making it well-suited for ultra-thin barrier films, interfacial bonding layers, and conformal coatings on textured or nanoscale surfaces [44]. Although ALD is typically less rapid and more expensive than PVD, its ability to precisely control thickness and achieve conformal coatings is beneficial for multifunctional coatings in modern glazing applications [44,45].

Sol–gel synthesis is a technique that lies in between chemical techniques and solution techniques that involves hydrolysis and condensation of metal alkoxides or inorganic salts to develop oxide or hybrid matrices under relatively low temperatures [22,26,46]. Sol–gel coatings on glass and polymeric substrates by dipping, spinning, or spraying of sol–gel suspensions are capable of producing anti-reflective, self-cleaning, and scratch-resistant films after appropriate drying and thermal treatments [22,46,47]. In addition to the variety of materials available through sol–gel synthesis, one may introduce nanoparticles, organic species, and functional groups [22,46,47].

### 3.3. Solution and Polymer Processing (Spray, Slot-Die, Roll-to-Roll, Inkjet)

Coatings fabricated through solution-based processing techniques such as spray coating, slot-die coating, roll-to-roll (R2R) coating, and inkjet printing provide several benefits when deposited for large-scale greenhouse windows because they are scalable and compatible with flexible substrates [38,48]. The spray coating method allows the application of functional suspensions or polymer solutions on complicated surfaces and is commonly used in depositing hydrophilic, hydrophobic, or photocatalytic coatings consisting of TiO_2_, SiO_2_, and other combinations [48,49]. Slot-die and R2R coating technologies are capable of continuously depositing uniform coatings on continuously fed web-like materials, making them suitable for polyethylene (PE) or polymer composite films in greenhouses [38,48].

The inkjet printing process offers digital patterning, allowing spatial regulation of coating composition and thickness without the need for masks. Such a property is highly beneficial for embedding optical filters, self-cleaning zones, or functional circuits within glazing [50]. In all solution-based approaches, solvent selection, rheological properties, wetting characteristics, and drying mechanisms significantly impact coating uniformity, defect generation such as pinhole formation and coffee-ring phenomena, and microstructural development [48,49,50]. In the case of polymeric binder systems or hybrid films, photopolymerizable resins are typically used to ensure fast cross-linking and minimal thermal expansion of the substrate [49,50].

### 3.4. Post-Treatments and Microstructure Control

The post-deposition treatments such as thermal annealing, ultraviolet curing, laser treatment, and plasma processing play crucial roles in improving the structure and properties of optical and self-cleaning coatings [46,51]. Thermal annealing densifies sol–gel and sputtered films, promotes the crystal structure formation in oxide films, and triggers photocatalytic and thermochromic responses (e.g., TiO_2_ or VO_2_). Nevertheless, such approaches should be meticulously coordinated with the thermal stability differences between glass and polymeric substrates [46,51,52]. Ultraviolet curing of acrylate and epoxy-based composites facilitates fast cross-linking under ambient conditions and increases their strength and stability without significant thermal stress [49,53].

Table 2 provides a comprehensive comparison of technique suitability for greenhouse glazing production.

**Figure 4:**
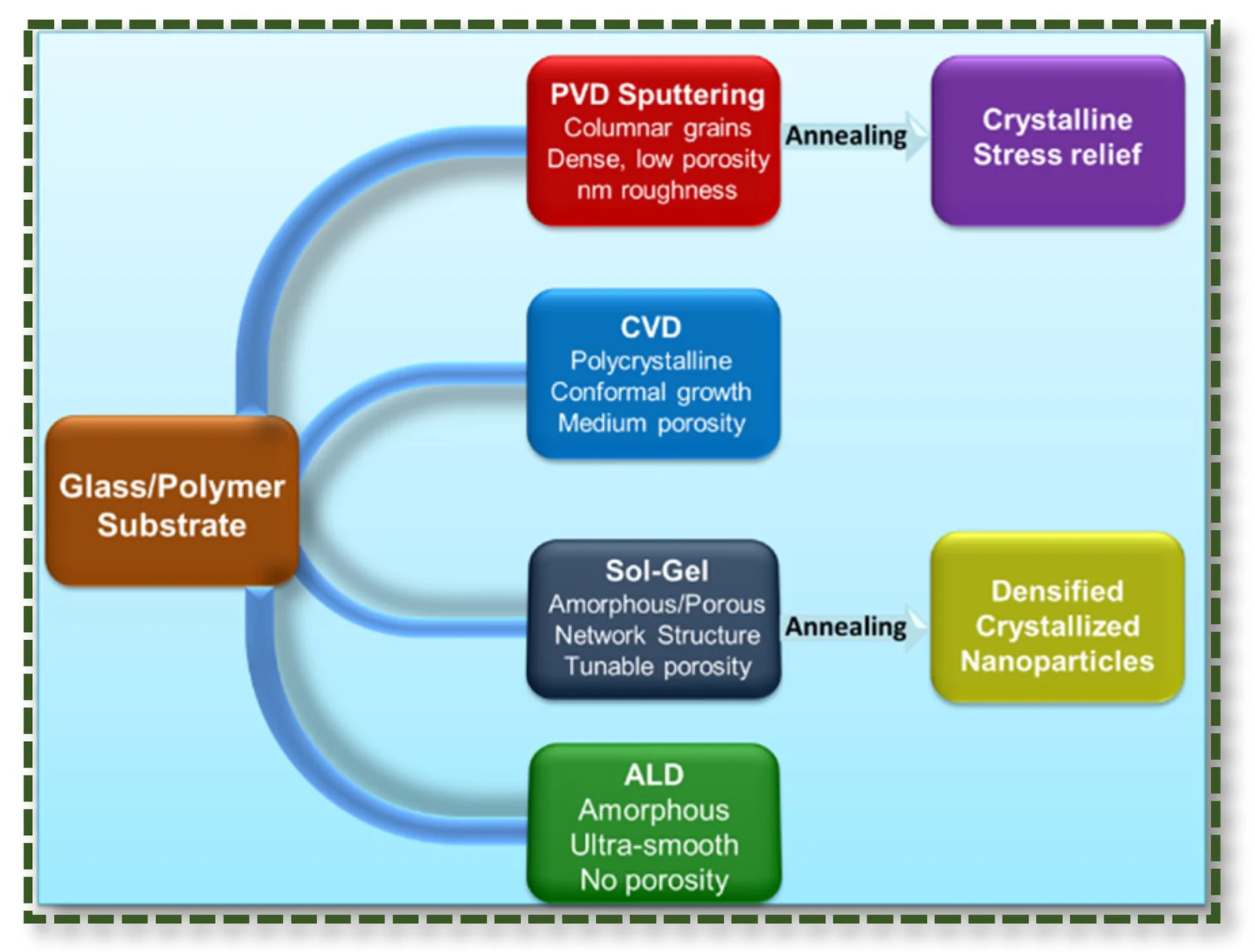
Microstructure evolution by deposition technique.

Various plasma treatments (oxygen, nitrogen, and argon are some examples) can be used to alter the surface chemistry and topology at the substrate level by increasing surface coating adhesion, changing surface wettability (hydrophilic or hydrophobic nature), and cleaning any organic surface contamination before deposition [51,54]. Laser techniques and localized heating can be used in patterning or local crystallization of thin films in order to create graded or pixilated functionality such as patterned wetting and changing optical properties [52,54]. Grain size, porosity, interfacial condition, and residual stress can all be tailored using appropriate combinations of the post-treatment methods mentioned above and this will directly have an impact on the coating characteristics presented in the next sections [46,51,52,53,54]. These effects are directly linked to the differences in microstructure (Figure 4) that occur depending on the fabrication process used, either PVD or sol–gel.

## 4. Structure–Property Relationships

However, for the optimal performance of optical coatings, including self-cleaning ones, their atomic composition, microstructure, and macroscopic properties need to be correlated with each other [55,56]. In the case of greenhouse glazing, the simultaneous presence of features such as high transmission of PAR radiation, selective near-infrared rejection, self-cleaning abilities, and resistance to environmental impact makes these correlations especially important [55,56,57]. The following section examines the relationship between coating composition, crystal structures, and morphology and the optical properties of interfaces, surface wetting behavior, thermal emittance, and mechanical stability of the materials in question.

### 4.1. Optical Properties: Refractive Index, Band Gap, Interference Design, Spectral Selectivity

Optical properties in transparent coating films are mostly controlled by their electronic structures, refractive index difference, and interference mechanisms in multilayers [55,58]. Large band gap oxides like TiO_2_ (3.0–3.2 eV), ZnO (3.3–3.4 eV), and SiO_2_ (>9 eV) exhibit transparency for visible and PAR while selectively absorbing or reflecting UV and NIR through absorption thresholds or free carriers [58,59]. Dopant techniques like aluminum doping to ZnO and fluorine doping to SnO_2_ control the number and movement of carriers, thus exhibiting low-emissive properties while having luminous transmittance greater than 80% [59,60].

In multilayer dielectric stacks, accurate manipulation of the refractive index of individual layers (n SiO_2_ ≈ 1.46; n TiO_2_ ≈ 2.4–2.6) and layer thickness (λ/4 at the design wavelength) results in constructive interference in the PAR region (400–700 nm) and destructive interference in the NIR region (700–2500 nm), resulting in solar heat gain coefficient (SHGC) values as low as 0.3–0.4 [56,61]. The gradual refractive index profile at the interface, possible using co-sputtering techniques or multilayer structures, reduces reflection losses, thus improving broadband anti-reflective properties that are important to maximize light delivery to greenhouse plants [61,62]. Surface texturing or porosity also increases light scattering for greater canopy penetration, all the while keeping high transmittance values [62].

In greenhouse environments, the optical design of coatings must be optimized according to crop-specific spectral requirements rather than solely minimizing solar heat gain as commonly practiced in architectural glazing. Since PAR radiation (400–700 nm) directly drives photosynthesis, maintaining transmittance above approximately 80–85% is generally desirable to avoid reductions in crop growth and yield. Meanwhile, selective attenuation of NIR wavelengths is advantageous because excessive thermal loading can increase greenhouse temperatures, induce plant stress, and elevate cooling or ventilation demands. Several studies have demonstrated that spectral-selective multilayer coatings capable of transmitting PAR while reducing NIR penetration can improve thermal regulation without significantly compromising plant productivity. Furthermore, the transmission of UV radiation must be carefully engineered because moderate UV-A exposure may enhance secondary metabolite production, coloration, and pest resistance in crops, whereas excessive UV-B exposure can negatively affect plant health and accelerate polymer degradation in greenhouse coverings.

Quantitative structure–property correlations for leading greenhouse coating systems are summarized in Table 3.

**Figure 5:**
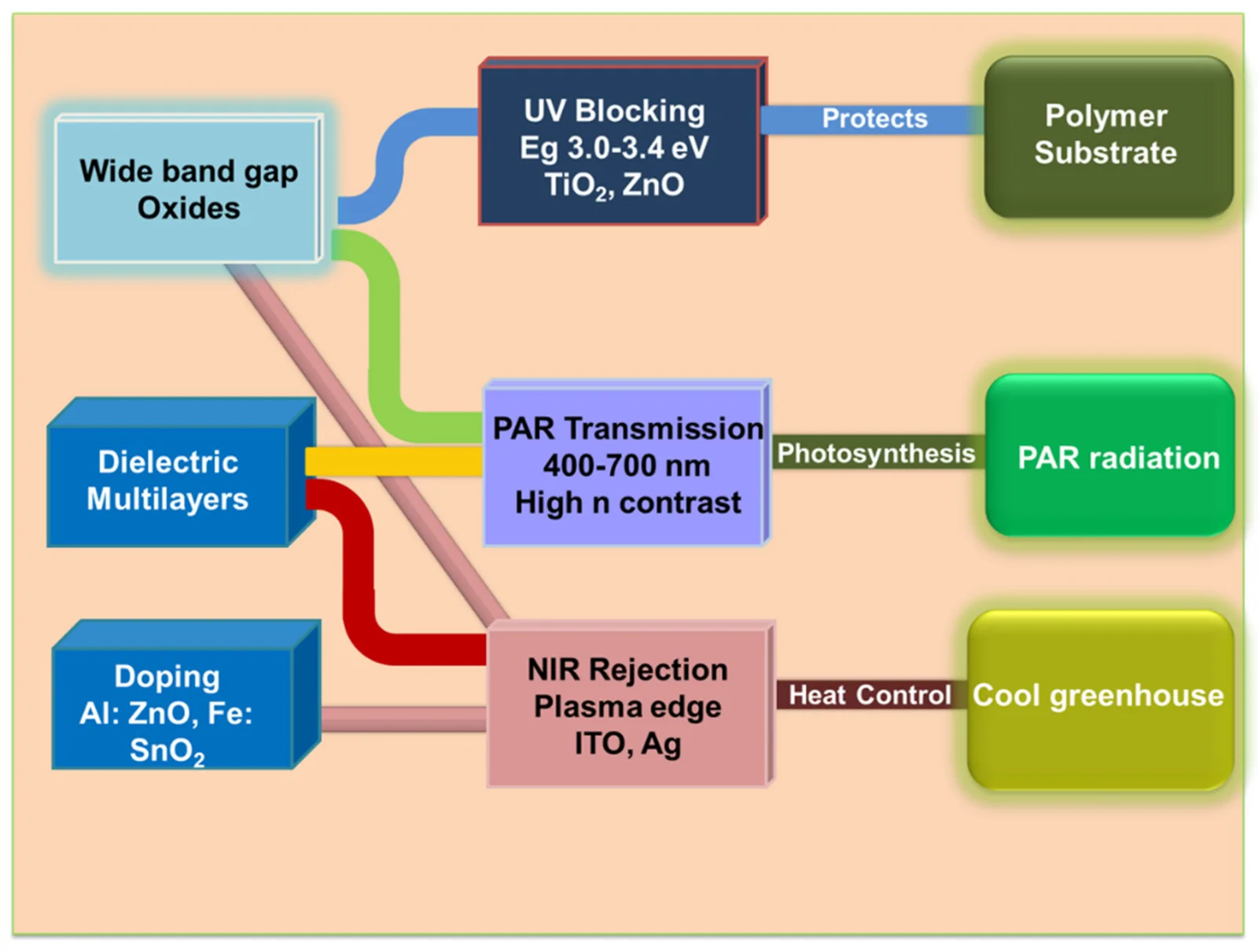
Optical property tuning mechanisms.

Figure 5 illustrates the specific mechanisms achieving spectral selectivity, with wide band gap oxides blocking UV while multilayer interference optimizes PAR versus NIR transmission.

#### Chemical Composition and Bonding as Drivers of Coating Function

The chemical composition of a functional coating determines its electronic structure, bonding characteristics, surface reactivity, and interaction with the underlying substrate and therefore represents a fundamental level of control over its optical and interfacial performance. In wide-band-gap oxide coatings such as TiO_2_, ZnO, and SiO_2_, metal–oxygen bonding and the resulting electronic structure determine the absorption edge and visible-light transparency. Modification of cation composition or introduction of aliovalent dopants can alter defect populations and carrier concentration, thereby affecting refractive index, electrical conductivity, free-carrier absorption, and infrared reflectance. For example, Al incorporation into ZnO and F incorporation into SnO_2_ can modify carrier concentration and infrared optical response, making these materials relevant to spectrally selective and low-emissivity coatings.

In photocatalytic TiO_2_ coatings, chemical composition and crystal phase are closely related to surface reactivity. Anatase TiO_2_ generally exhibits high photocatalytic activity because photogenerated charge carriers participate in surface redox reactions that promote the decomposition of adsorbed organic contaminants and surface hydroxylation, contributing to superhydrophilic behavior. Thus, photocatalytic and wetting properties are closely associated with the chemical and crystallographic state of the oxide surface.

In Ag-based low-emissivity multilayers, the metallic layer provides strong infrared reflection, whereas surrounding dielectric layers control optical interference, protect Ag from chemical degradation, and influence the optical response of the multilayer. Similarly, the thermochromic behavior of VO_2_ originates from its temperature-dependent electronic and structural transition, while dopants and compositional modifications can alter the transition temperature and optical modulation. These examples demonstrate the direct connection between chemical composition, phase stability, and optical functionality.

For sol-gel-derived coatings, hydrolysis and condensation reactions determine the development of the inorganic network, while precursor chemistry, solvent composition, catalyst concentration, drying, and thermal treatment influence porosity, hydroxyl content, shrinkage, and cracking. Organic–inorganic hybrid systems introduce additional chemical functionality but may also be more susceptible to UV-induced degradation. Therefore, coating chemistry must be considered together with processing conditions to obtain the required microstructure, functional properties, and long-term stability.

### 4.2. Surface Wetting and Self-Cleaning: Roughness, Chemistry, Hydrophilic vs. Hydrophobic Designs

Self-cleaning properties for coatings applied to greenhouses can be attained via a superhydrophobic state (contact angle θ > 150°) or a superhydrophilic one (θ ≈ 0°) of the coating surface, depending on the combination of topographical features and surface properties at a nanoscale level [67,69]. Superhydrophobic surfaces require hierarchical surface structures combined with surface energies lower than that of water, which can be accomplished using fluorosilane or hydrocarbon chemistries [69]. Nanometric-sized SiO_2_ particles incorporated into PDMS materials and plasma-etched silica films provide self-cleaning surfaces with contact angles exceeding 160° and roll-off angles of less than 5° on glass substrates [67,68].

**Figure 6:**
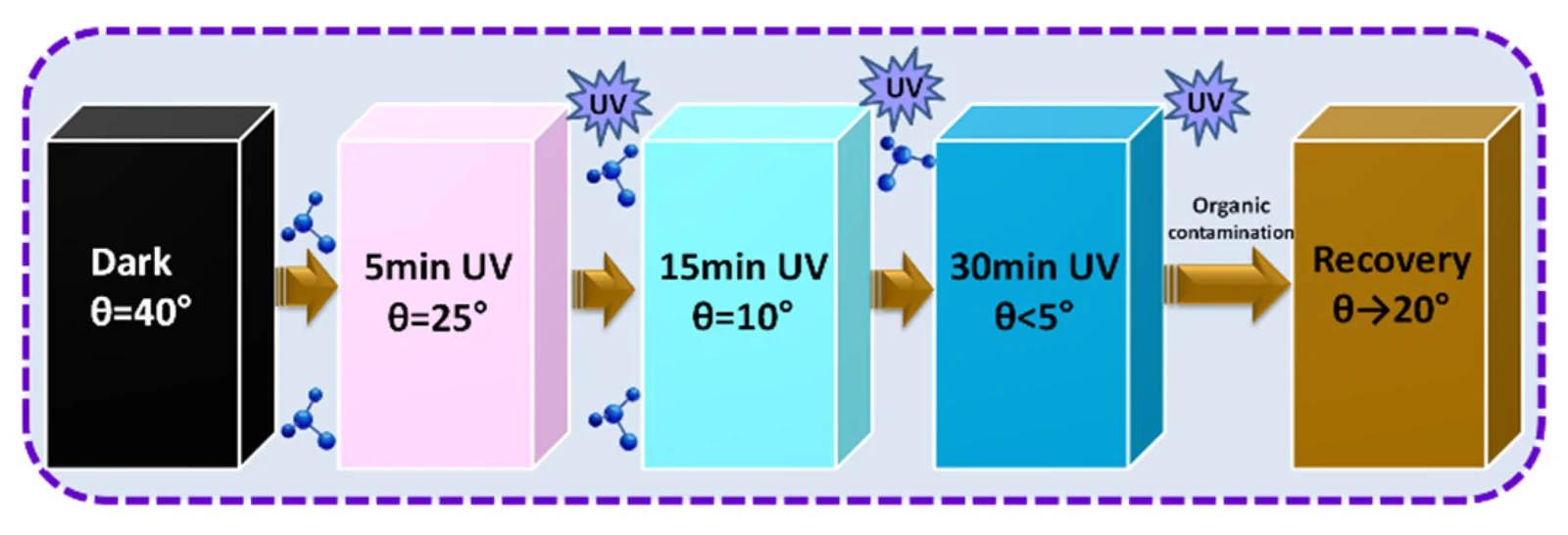
TiO_2_ contact angle evolution (From Ref. [63]).

In contrast, superhydrophilic surfaces facilitate full water sheeting through nanoscale surface roughening on high-surface-energy substrates, usually aided by photocatalytic TiO_2_, which continuously breaks down organic deposits using UV light [63]. The anatase form of TiO_2_ has a pronounced capacity for hydroxyl groups and hole trapping, leading to superhydrophilicity (θ < 5°), which is stable across repeated cleanings [63]. Ensuring the stability of both states in response to wear, UV degradation, and hydrocarbon fouling presents a significant difficulty, as the progressive reduction in surface chemistry or physical structure reduces self-cleaning performance during greenhouse operating periods [63,68]. The development of photocatalytic superhydrophilicity on TiO_2_ surfaces under UV radiation is illustrated in Figure 6.

### 4.3. Thermal and Emissivity Control: Low-E Stacks, IR-Reflective Coatings

Low-E coatings reduce radiative thermal transfer by reflecting long-wave infrared (LWIR) radiation while maintaining high transmission of the desired solar wavelengths [4]. Representative low-E multilayers employ one or more thin metallic Ag layers separated and/or enclosed by dielectric layers, such as dielectric/Ag/dielectric or dielectric/Ag/dielectric/Ag/dielectric architectures. The Ag layer provides the principal infrared-reflective and low-emissivity function, whereas the surrounding dielectric layers control optical interference, visible-light transmission, and spectral selectivity and also protect the Ag layer against oxidation, corrosion, and mechanical degradation [4,64]. The resulting optical and thermal performance therefore depends on the thickness, continuity, optical constants, and interfacial quality of both the metallic and dielectric layers [64,70]. Transparent conducting oxides such as ITO and FTO can provide infrared control with greater chemical stability, although their higher electrical sheet resistance generally leads to weaker infrared selectivity than highly conductive Ag-based layers [64,70].

The thermochromic coating of VO_2_ allows for variable emissivity regulation, going from the low-emissivity state (ε ≈ 0.4) to the high-emissivity state (ε ≈ 0.8) upon heating through its thermochromic transition temperature (≈68 °C, with the transition temperature potentially reduced toward 40–50 °C through appropriate doping) [65]. This phase change entails significant near-infrared reflectance modulation (ΔTsol > 10%), indicating the potential of VO_2_-based coatings for adaptive solar and thermal control in glazing applications [65,66]. For greenhouse glazing, however, their practical effectiveness depends on achieving an appropriate transition temperature while maintaining adequate PAR transmission, solar modulation, and long-term durability.

### 4.4. Mechanical Properties: Hardness, Adhesion, Abrasion Resistance, Erosion Behavior

Outdoor glazing is subjected to mechanical stresses arising from airborne particles transported by wind, hail impact, repeated cleaning, handling, and temperature-induced expansion and contraction [71]. Hardness (H), commonly measured by nanoindentation, provides an important indication of resistance to indentation and scratching, although it does not by itself determine the overall wear or erosion resistance of a coating. Reported hardness values vary substantially with composition, deposition method, crystallinity, porosity, densification, heat treatment, and indentation conditions. Representative sol-gel-derived oxide coatings can exhibit hardness values of approximately 1–5 GPa, whereas dense and highly crystalline oxide films produced by other deposition routes can reach values above 10 GPa [71,72]. Adhesion at coating/substrate interfaces, evaluated using tape, pull-off, or scratch tests through parameters such as the critical load (L_c_), is equally important because poor interfacial bonding can lead to cracking and delamination during mechanical or environmental exposure. Graded interfaces and adhesion-promoting interlayers can therefore improve coating integrity and resistance to delamination [72,73].

Abrasion and erosion involve different mechanical damage mechanisms and should therefore be evaluated separately. Abrasion primarily results from repeated rubbing, sliding, or contact with cleaning tools and particulate matter, whereas solid-particle erosion results from repeated impact of airborne or transported particles on the coating surface. The resistance of a coating to these processes depends on multiple interrelated factors, including hardness, fracture toughness, elastic properties, microstructure, surface roughness, coating thickness, and coating/substrate adhesion. Erosion behavior is additionally influenced by the size, hardness, shape, and velocity of the impacting particles, as well as the impact angle and exposure conditions. Therefore, hardness alone cannot be used as a universal predictor of abrasion or erosion resistance, and the relative importance of these parameters depends on the coating composition, microstructure, and specific damage mechanism.

For transparent greenhouse glazing, mechanical degradation should also be evaluated in terms of its optical and functional consequences. Surface abrasion or particle erosion can increase surface roughness and haze, reduce visible and PAR transmission, and impair functional surface properties, such as photocatalytic activity and hydrophobicity, even before substantial coating loss occurs. Accordingly, abrasion and erosion resistance should be assessed under representative service conditions, with the particle characteristics, impact velocity and angle, exposure duration, and cleaning conditions clearly specified. Changes in optical transmission, haze, adhesion, surface roughness, and functional properties can then be used to assess the extent of degradation. Residual stresses generated during deposition or thermal treatment should also be considered because excessive tensile or compressive stresses can promote cracking, delamination, or failure during thermal cycling [74].

### 4.5. Coupling Between Microstructure and Performance

The microstructure–property relationships discussed above originate from the underlying chemical characteristics of the coating materials. Variations in composition, bonding environment, oxidation state, dopant concentration, defect population, and surface functional groups can influence nucleation, crystallization, grain growth, porosity, and interfacial bonding during deposition and post-treatment. These chemically controlled structural features subsequently affect light absorption and scattering, charge-carrier behavior, surface hydroxylation and wettability, thermal emissivity, and mechanical stability. Therefore, the relationship can be considered as a continuous sequence of chemical composition and bonding-phase and defect structure-microstructure and interfaces-functional properties-greenhouse performance and durability.

Microstructure is the fundamental determinant of all functional characteristics due to interconnected influences on light scattering, carrier mobility, surface activity, and mechanical strength [35]. Grain growth in columnar form within sputtering films induces birefringence, but inter-columnar voids may occur, leading to water absorption and delamination [22,35]. Porosity in sol–gel layers, which enhances photocatalytic surface area and hydrophilicity, causes high scattering losses and moisture absorption unless carefully managed [22,75].

**Figure 7:**
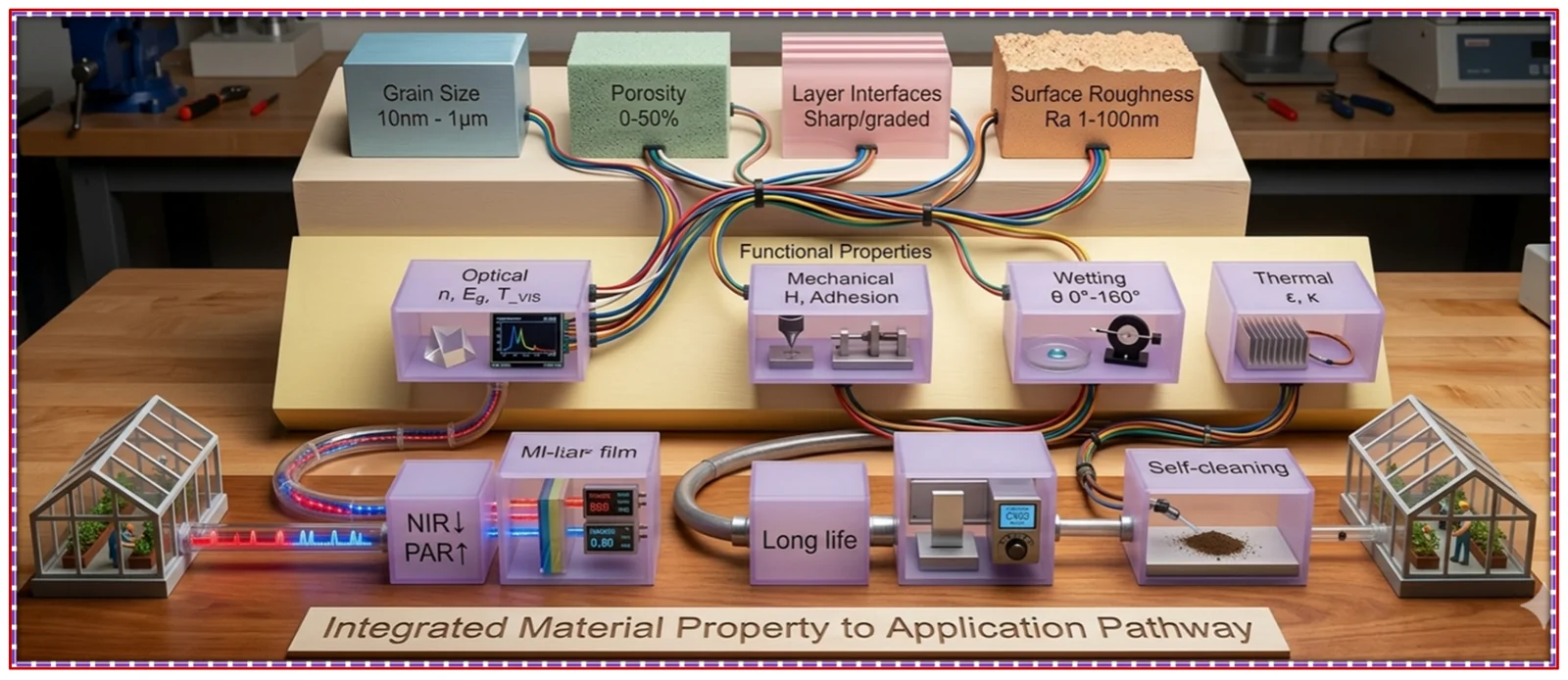
Structure–property map for greenhouse coatings.

**Figure 8:**
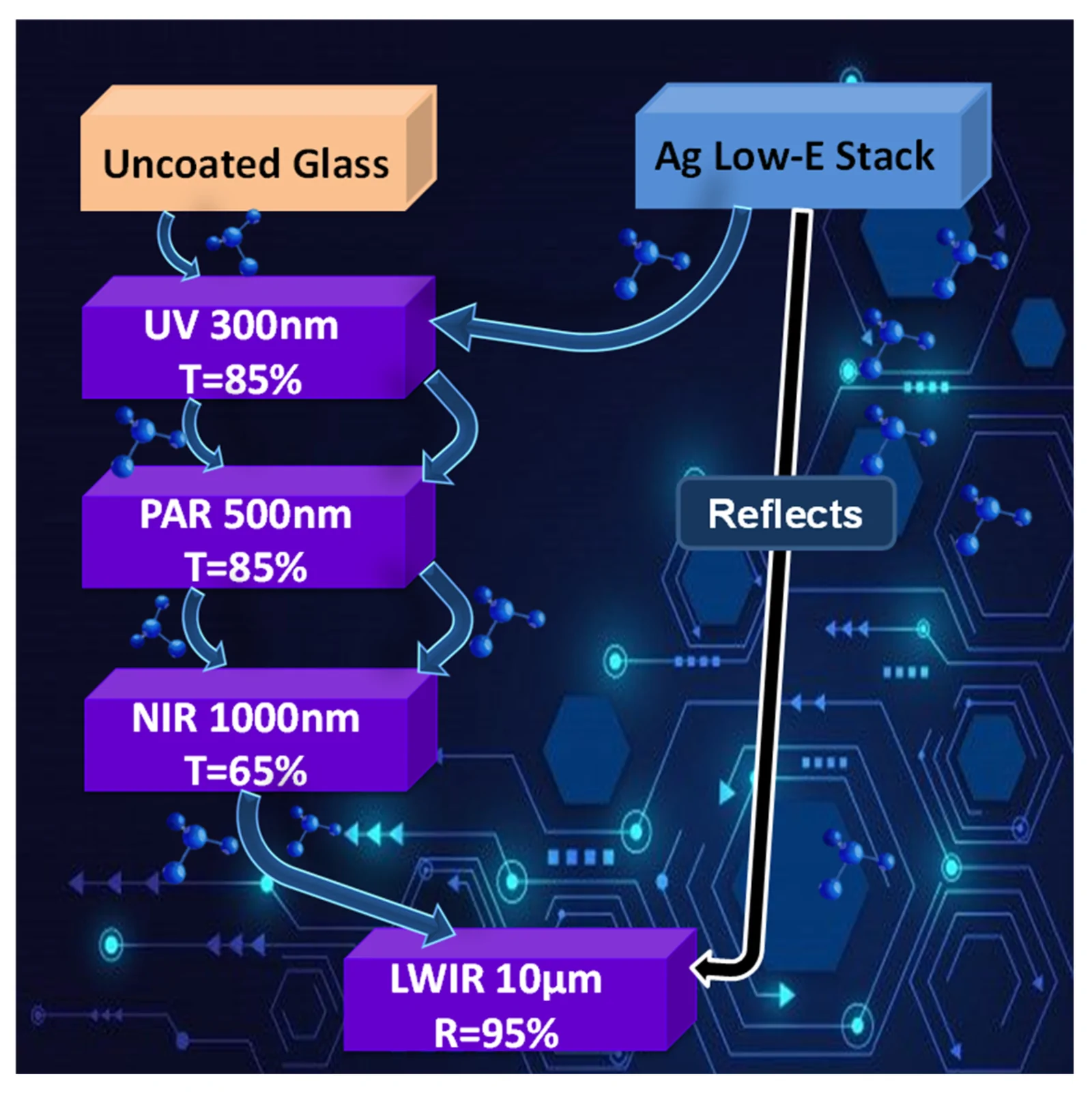
Low-E spectral data (From Ref. [56]).

The presence of grain boundaries in polycrystalline oxides leads to a decrease in the efficiency of the photoreaction and an increase in thermochromic switching speed and acts as a fast diffusion channel for alkali ions originating from soda-lime glass and able to contaminate the silver-based low-emissivity coating [75,76]. The quality of the interfaces between each layer within the multilayered system defines the filter efficiency and development of stress due to temperature cycling [76]. Moreover, the hierarchy of nanostructures contributes to better wetting by creating dual-scale roughness, improves the light diffusion effect by creating forward-scattering effects, and increases mechanical adhesion through increased surface area [76,77]. The basic connection between the microstructure characteristics and the functional characteristics is shown in Figure 7, where the influence of grain size, porosity, and surface roughness on the transmission of light, wettability, mechanical stability, and heat management is demonstrated. The spectral selectivity of the low-emissivity silver layer is compared to the bare glass in Figure 8.

At the coating/substrate interface, chemical compatibility and interfacial bonding strongly influence adhesion and environmental stability. Hydroxylated glass surfaces can interact with silanol-containing sol–gel precursors, while plasma or other surface-activation treatments can increase surface energy and improve wetting of the coating precursor. In contrast, polymeric substrates generally possess lower surface energy and different thermal expansion behavior, making interfacial adhesion more challenging. Consequently, the selection of adhesion-promoting interlayers, surface functionalization, and graded interfaces becomes particularly important for polymer-supported greenhouse coatings.

## 5. Durability and Environmental Stability

The long-term suitability of greenhouse glazing coatings depends on their ability to maintain optical, self-cleaning, and mechanical performance under the combined influences of ultraviolet irradiation, thermal cycling, mechanical abrasion, moisture ingress, and chemical exposure over the typical service lifetime of commercial greenhouses (10–20 years) [58,78]. Unlike conventional architectural glazing, greenhouse coatings operate under more complex environmental conditions because of continuous exposure to irrigation-induced wetting, elevated humidity, agrochemical residues, airborne soil particles, repeated cleaning, and intense solar radiation [78,79]. Under these conditions, multiple degradation mechanisms can act simultaneously, progressively deteriorating the optical properties, self-cleaning ability, adhesion, and mechanical durability of the coating and ultimately compromising its functional performance [58,80]. Therefore, durability evaluation of greenhouse coatings should extend beyond conventional single-factor laboratory aging tests and consider environmental conditions representative of agricultural operation, including persistent humidity, agrochemical contamination, repeated cleaning, mechanical abrasion, thermal cycling, and long-term UV exposure.

Because these stressors can interact during service, combined or sequential accelerated-aging protocols can provide a more realistic assessment of coating durability than isolated exposure to individual factors. A representative testing strategy could combine controlled UV irradiation with elevated humidity or condensation, thermal cycling, and periodic exposure to relevant agrochemical residues, followed by evaluation of changes in visible and PAR transmission, haze, surface wettability, chemical structure, adhesion, mechanical integrity, and other coating-specific functional properties. Such multi-stressor testing is important because interactions between UV radiation, moisture, temperature fluctuations, mechanical damage, and chemical contaminants may accelerate degradation or produce failure mechanisms that are not evident in single-factor tests. However, the intensity, duration, sequence, and frequency of each stressor should be selected according to the target greenhouse environment, coating chemistry, substrate, and intended service conditions since a single universal combined-aging protocol is not applicable to all greenhouse glazing systems.

### 5.1. UV-Induced Degradation and Photo-Corrosion

The mechanism for ultraviolet light-induced coating failure occurs mainly via photochemical cleavage of chemical bonds, carrier production, and catalysis at the surface [81]. In TiO_2_ and ZnO films exhibiting photocatalytic properties, the extended use of UV light results in the degradation of superhydrophilic properties owing to the degradation of hydroxyl groups and defects responsible for the superhydrophilic property, such that the contact angle gradually increases from <5° to between 20° and 40° over 1000 to 5000 h of QUV tests [81,82].

The organic molecules in sol–gel hybrid and polymer systems undergo accelerated photodegradation, wherein C-H, C-O, and Si-C bonds break to produce volatiles, carboxylic acids, and yellowing compounds that cause light scattering and reduced PAR transmission [83,84]. Fluorinated surface chemistries can undergo chemical changes under severe environmental exposure, and their long-term stability and potential release of fluorinated species should therefore be evaluated alongside their persistence and environmental implications [84]. In the metallic low-emissivity films, UV scattering by the oxide film can oxidize the silver layer, resulting in increased sheet resistance and emissivity [85].

### 5.2. Moisture, Temperature Cycling, and Mechanical Loading

The hydrothermal aging process involves using moisture and the effects of temperature to increase delamination and crack formation rates [58,86]. Moisture enters through the columnar voids present in PVD layers and connected pores in sol–gel layers and leads to hydrolysis of the Si-O-Si network and metal oxide bonds, as well as promoting alkali ion diffusion from the soda-lime glass substrate [86,87]. Differences between the thermal expansion coefficients of the coating material and the glass substrate can generate biaxial tensile or compressive stresses during thermal cycling, potentially promoting cracking or interfacial decohesion.

Erosion of the protective coating due to hail impact (hailstones of 5–20 mm in diameter and 10–30 m/s in impact velocity), erosion by wind-transported dust particles (particles of 10 to 50 μm SiO_2_ in diameter and 5 to 20 m/s in velocity), and mechanical abrasion during cleaning are yet more factors deteriorating coating quality [88,89]. The initial cracks form on surface defects and further spread via fatigue processes; the rate and extent of crack initiation and propagation depend on coating thickness, residual stress, hardness, toughness, microstructure, and interfacial adhesion [89].

**Figure 9:**
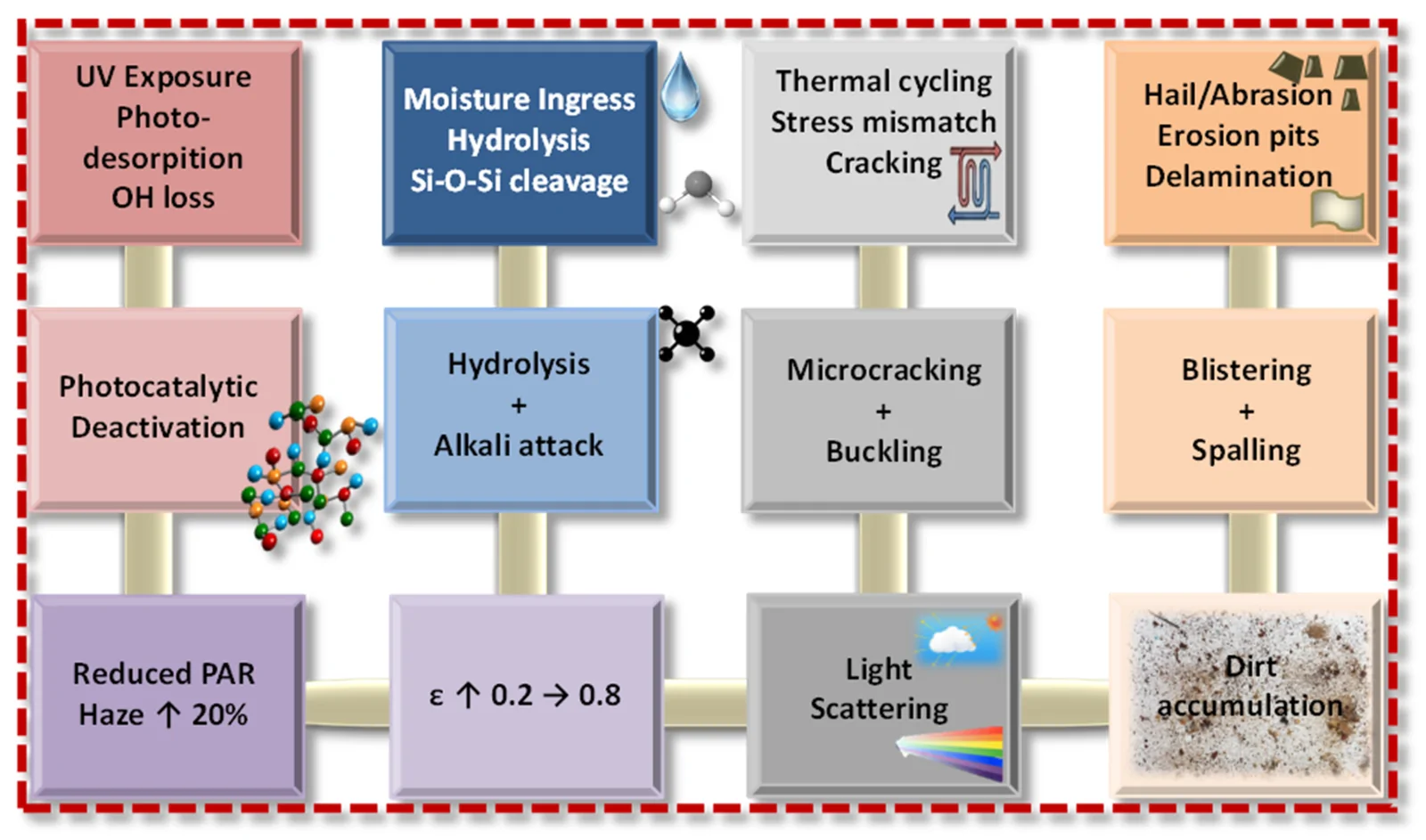
Primary failure mechanisms in greenhouse coatings.

Degradation routes that influence greenhouse coatings are shown in Figure 9, where self-cleaning properties become deactivated due to UV radiation while moisture-induced hydrolysis and physical damage further decrease PAR transmission.

### 5.3. Failure Modes: Delamination, Cracking, Yellowing, Loss of Functionality

Failure due to coating takes place by various interconnected mechanisms leading to deterioration of the performance of the greenhouse [90]. Delamination is caused by blister formation due to separation between the coatings at edges where water enters or when hit by hailstones and results in light scattering as well as accelerated corrosion of the substrate [87,90]. The cohesive cracks are a result of film stresses exceeding the material’s fracture toughness [87].

Yellowing due to conjugated carbon compounds (λ_max_ = 350–450 nm) causes loss of available PAR by 5–15% over 2–3 years, whereas the loss of superhydrophilicity or hydrophobic properties leads to the deposition of dirt, which may lead to up to a 20–50% decrease in transmittance within a few months [91]. Metal-based coatings with low-E values exhibit emissivity changes from 0.04 to greater than 0.2, which results in the loss of winter-time heat retention effects [91]. The thermochromic properties of VO_2_ undergo a reduction of switching contrast (ΔT_sol_) from more than 10% to less than 3%, caused by a blunting of the metal–insulator transition because of grain growth and defect accumulation [92,93].

### 5.4. Standardized Testing and Accelerated Aging Protocols

For accurate measurement of coating resistance, standard tests simulating the effects of climate in the greenhouse need to be established [94]. The ASTM G154/G155 QUV test method for 340 nm light source irradiation with temperature cycling from 40 to 60 °C and exposure time between 200 and 4000 h examines UV resistance through color change (ΔE*), haze growth, and evolution of the contact angle [94,95]. Thermal cycling test (from –20 °C to +60 °C, up to 1000 cycles) evaluates adhesion failure and cracking by means of tape tests (ASTM D3359) and nano hardness critical load (Lc) [95]. Taber wear test (with CS-10F wheels, 1000 cycles, haze change < 5%) and sand erosion testing (ASTM D968) quantify mechanical robustness [95,96].

Salt mist (ASTM B117, 35 °C, 5% NaCl, 1000 h) and chemical resistance tests (simulated acid rain, agro-chemicals) evaluate corrosion mechanisms appropriate for regions with a coastal environment or intensive agricultural practices [96]. Accelerated exposure to weathering (e.g., ISO 11341 Xenon Arc Lamp, 3000 kJ/m^2^) gives realistic estimates of lifetime when calibrated against outdoor exposure data; however, the interaction effects of stressors are difficult to predict and simulate [96,97]. Outdoor testing using a rack system in greenhouses is an ideal method, which takes about 2–5 years of exposure [97].

Such protective techniques, like dense barrier layers (ALD Al_2_O_3_), self-healing chemistry, and adaptive multilayer systems with sacrificial top coats, have been found to be particularly promising for achieving commercial lifetime expectations in extreme greenhouses [98].

**Figure 10:**
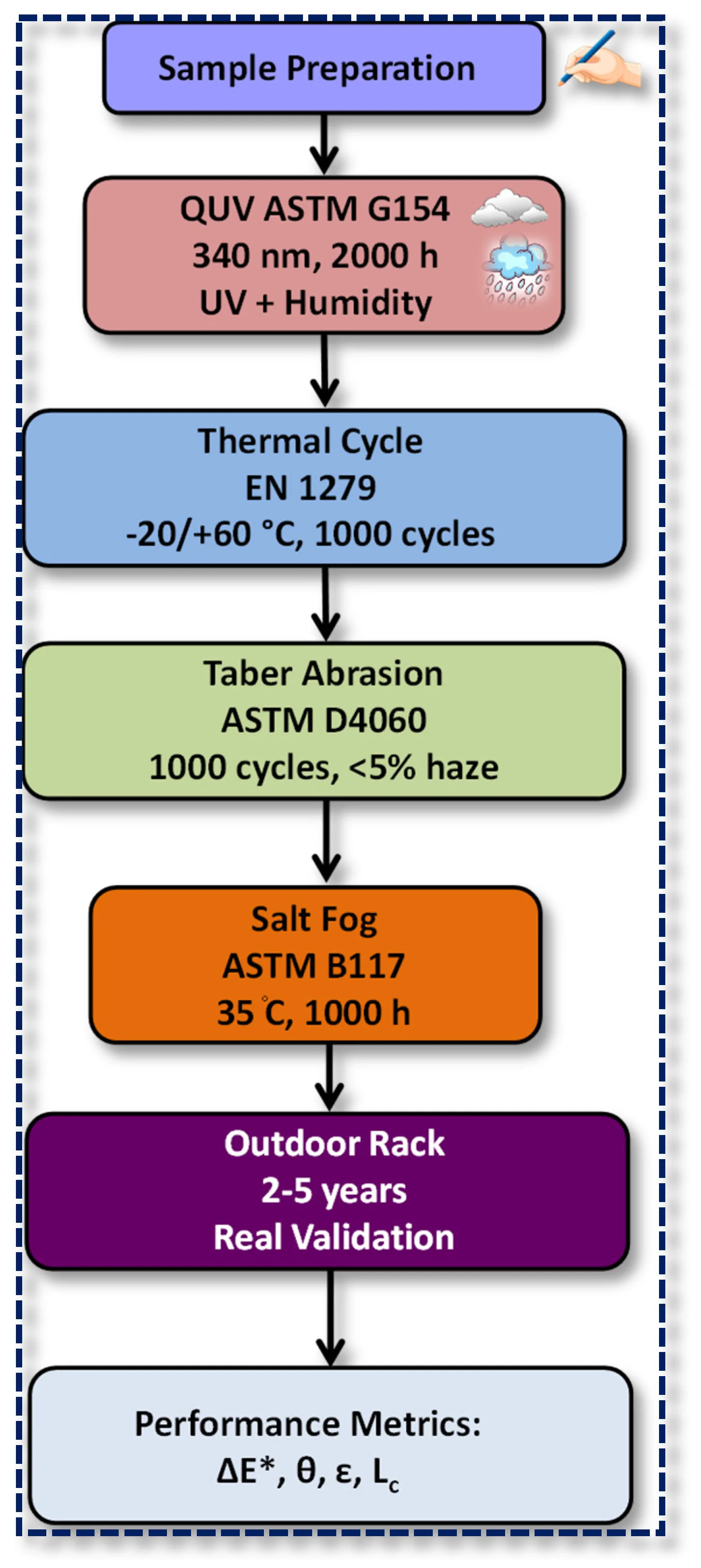
Accelerated aging test sequence.

Accelerated weathering test procedures that include QUV exposure, thermal cycles, abrasion, and salt fog (Figure 10) can estimate 10–20 years of outdoor exposure.

Table 4 details the standardized metrics and pass criteria for each durability test relevant to greenhouse conditions.

## 6. Application Case: Coatings for Greenhouse Glazing

Coatings used on greenhouse glazing have to convert their physical characteristics into tangible performance advantages when taking the operational realities into account [99,100]. Unlike architectural windows, the PAR transmission properties of greenhouse glazing, its spectrum manipulation capabilities, and self-cleaning abilities are all subject to more rigorous requirements, since minor differences in the amount of light reaching crops and contamination of the surfaces may severely affect the efficiency of plant growth and productivity [99,101]. This sub-section considers how coating development strategies are applied in the case of greenhouses, concentrating on optical design for agriculture, surface functionality in greenhouse climates, energy and longevity factors, and general glazing technology issues.

### 6.1. Optical Design for Crops: PAR Transmission vs. NIR Rejection, UV Management

The spectral requirements of greenhouse crops vary depending on species, developmental stage, and climatic conditions, making coating optimization more complex than in conventional building glazing. Crops such as tomatoes, cucumbers, and leafy vegetables generally require high PAR availability for biomass production, whereas excessive heat accumulation due to NIR transmission can induce physiological stress, reduce fruit quality, and increase transpiration rates. Consequently, greenhouse coatings should not simply maximize total visible transmittance but instead selectively regulate spectral regions in a manner that balances crop productivity, thermal comfort, and energy efficiency.

A key design criterion of optics for coatings used in greenhouses is to achieve maximum possible transmission and effective distribution of PAR waves (400–700 nm) while restricting high amounts of NIR waves (700–2500 nm) that generate excessive heating and balancing the amount of UV radiation [100,102]. Multilayer and low-E coatings currently being used in building glazing can also be effectively used in greenhouses through modifying them according to the desired wavelengths and optimal angle of incidence, thereby maintaining an adequate PAR wave transmission level of ≥80–85%, along with minimizing solar heat gain coefficients sufficiently to cut down the need for summertime cooling or ventilation [102,103]. Diffuse reflective coating and surface micro/nanotechnology allow even deeper light penetration through the vegetation layer of the canopy, ensuring an equal level of photosynthetic activity and preventing excessive heat and burns to the upper part of the canopy [101,104].

The issue of protection against UV radiation in greenhouses is much more complex than in buildings: UV-A radiation allows some plants to develop their secondary metabolism and coloring, whereas UV-B is harmful for all organic materials [101,105]. In this way, TiO_2_, ZnO, and oxide-based coatings are engineered to exhibit sharp rejection in the UV-B range while permitting some controlled passage of UV-A radiation, optimizing the physiological effects on plants while ensuring polymer substrate integrity and minimal pest behavior [105,106]. Thermochromic materials, such as VO_2_ and other phase change oxides, provide yet another optical dimension through dynamic suppression of NIR radiation above certain temperatures, which may be adjusted close to greenhouse thermal comfort levels (i.e., 30–35 °C), effectively avoiding midday thermal stress while preserving PAR radiation [103,107].

#### Relationships Between Spectral Performance and Plant Physiology

The optical performance of greenhouse glazing should ultimately be evaluated in terms of its effects on plant physiological processes rather than solely by transmission or rejection values. PAR (400–700 nm) provides the primary photon flux for photosynthesis, and reductions in transmitted PAR can decrease leaf photosynthetic rate and the photosynthetic daily light integral available to the crop, particularly under low-light or winter conditions. Consequently, maintaining high PAR transmission is important for biomass accumulation and yield. However, the biological response is not determined only by the total PAR quantity. The spectral distribution within the PAR region also affects plant photoreceptors and photomorphogenesis. Blue and red wavelengths contribute strongly to photosynthetic activity, while blue light influences stomatal responses and plant morphology and red/far-red spectral balance regulates processes such as shade responses, stem elongation, and developmental transitions. Therefore, coatings that selectively modify the spectrum should be evaluated according to both photon quantity and spectral quality rather than visible transmittance alone.

NIR radiation (approximately 700–2500 nm) contributes primarily to radiative heating rather than directly driving photosynthesis. Excessive NIR transmission can increase leaf and canopy temperature, enhance transpiration and water demand, and increase greenhouse cooling requirements. Selective NIR rejection can therefore reduce thermal stress while preserving the PAR required for photosynthesis. However, excessive spectral filtering may also reduce the total radiation available to the crop or alter the greenhouse microclimate, indicating that NIR control should be optimized together with PAR transmission and environmental conditions rather than maximized independently. Studies of NIR-reflecting greenhouse covers have demonstrated this tradeoff, with different cover materials showing substantial differences in PAR transmission and consequent implications for crop growth and yield [108,109].

UV radiation also has physiological effects that should be considered in coating design. UV-A can influence plant morphology, pigmentation, secondary metabolism, and defense responses, whereas excessive UV-B can cause physiological damage and affect photosynthetic processes. Conversely, strong UV exclusion may reduce the accumulation of UV-responsive secondary metabolites such as phenolics and anthocyanins in some crops. Therefore, complete UV blocking should not automatically be considered optimal; the appropriate UV transmission window depends on crop species, developmental stage, and production objectives. Reviews of UV-blocking greenhouse coverings have reported species-dependent effects on photosynthesis, transpiration, growth, yield, and secondary metabolites [110].

Accordingly, the relationship between coating performance and crop response can be expressed as spectral transmission-photon availability and spectral quality-photosynthesis and photoreceptor signaling-plant temperature and water relations-biomass accumulation, yield, and quality. This relationship indicates that the optimum coating is not necessarily the one with the highest overall transmittance or strongest solar rejection but the one that provides a crop-appropriate spectral environment while simultaneously controlling excessive heat gain and maintaining stable greenhouse conditions. The optimal spectral design should therefore be considered crop-, season-, climate-, and growth-stage-dependent [111].

### 6.2. Self-Cleaning and Anti-Fog Coatings Under Greenhouse Operating Conditions

Self-cleaning and anti-fog properties have a direct impact on greenhouses’ productivity since they help keep the glass surfaces optically clear even in the case of condensation, dust accumulation, and biological deposits [112]. Superhydrophilic TiO_2_ coatings take advantage of photocatalytic effects in order to break down organic contamination while ensuring water sheeting without water droplets, thus reducing fogging inside greenhouses, where it is possible to wash off impurities using rainfall or irrigation spray [112,113]. On the contrary, superhydrophobic SiO_2_ or fluoropolymer coatings attempt to limit the contact of water with glass surfaces by facilitating water droplet rolling [113,114].

This combination of factors in the greenhouse, namely high humidity, large thermal excursions over the dew point temperature, and the use of fertilizers and pesticides, presents distinct issues with respect to the application of such coatings [102,114]. Photoactive surfaces may need enough UV radiation to activate the coating, while at the same time too much UV screening to protect the polymer structure may slow down their regeneration process; likewise, a hydrophobic top coat may become contaminated and eventually degrade due to exposure to agrochemicals [102]. Durable coatings designed for greenhouse applications include a strong inorganic bottom layer, which provides stability and chemical resistance, along with a renewable top layer for proper wetting characteristics [102,112].

### 6.3. Energy and Lifetime Considerations in Real Installations

Considering the energy implications, coating materials for the greenhouses have impacts on cooling, heating requirements, and lighting requirements, particularly those systems with artificial lighting and temperature controls [99,115]. It has been noted through experimental and modeling studies that using solar reflective or thermochromic coatings on the roof and side walls of a greenhouse can lead to a 15–30% reduction in peak cooling loads as long as PAR is not below certain thresholds for crops [102,107,115]. The use of low-E coatings during winter season or cold climates decreases radiative losses at night-time and thus reduces heating loads [103,116].

Economic considerations over a life-cycle revolve around the balance between the cost of the coating, energy savings, production increases, and maintenance cycles [115,117]. For instance, high-performance multilayer coatings on inflexible glass substrate that could last up to 15–20 years are economically viable for high-value climate-controlled plant production, whereas inexpensive coatings of shorter lifetimes may be economically feasible for polyethylene-covered tunnels and seasonally used greenhouses [117,118]. The degradation processes detailed in Section 5, UV exposure, abrasion, peeling, and wettability failure, inform such calculations and drive the inclusion of durability data in economic models for various types of greenhouses and climatic zones [116,117,118].

### 6.4. Comparison with Architectural and Solar Glazing Applications

The coating technology developed for greenhouse glazing is borrowed from architectural or solar energy applications; yet, the requirements and operational parameters vary significantly [103,119]. Architectural glass takes occupant comfort and glare as its primary considerations, and it usually sacrifices higher visible transmittance to achieve greater solar gain reduction, while the glazing for greenhouses needs to maintain stringent criteria on PAR transmittance and spectral distribution [101,103,119]. On the other hand, solar cells employ anti-reflective and self-cleaning coatings that maximize the light absorption in a narrower spectrum range [119,120].

These distinctions offer potential for leveraging mature coating stacks and deposition processes from construction and solar panel applications to greenhouse glazing, with further fine-tuning of the layers’ thickness, refractive index characteristics, and wetting behavior toward better horticultural efficiency [102,120]. On the other hand, the high standards set in terms of spectral selectivity and resistance to soiling in the case of greenhouse glazing could lead to the development of better designs for agricultural facades, integrated building agriculture, and transparent photovoltaic modules, where the coexistence of food and energy production is a reality [115,120,121]. The comparison between different glazing technologies is tabulated in Table 5.

## 7. Critical Comparison and Performance Tradeoffs

Although numerous coating technologies have been proposed for greenhouse glazing applications, their practical implementation is ultimately governed by competing requirements involving optical performance, durability, manufacturing complexity, and economic feasibility. Consequently, evaluating individual coating systems solely on the basis of laboratory performance may lead to misleading conclusions. A meaningful assessment requires critical comparison of different approaches and identification of the tradeoffs that govern practical deployment.

### 7.1. Superhydrophilic Versus Superhydrophobic Self-Cleaning Strategies

Self-cleaning coatings generally rely on either superhydrophilic or superhydrophobic mechanisms [63,67,68,69]. Superhydrophilic surfaces, typically based on TiO_2_ photocatalysis, promote continuous water-sheet formation and decomposition of organic contaminants, whereas superhydrophobic surfaces utilize hierarchical roughness and low surface energy to facilitate droplet rolling and particle removal [63,67,68,69].

Both approaches have demonstrated excellent laboratory performance; however, their practical durability differs significantly. Superhydrophilic coatings generally exhibit superior chemical stability and resistance to environmental degradation owing to the intrinsic robustness of oxide surfaces. In contrast, superhydrophobic coatings often depend on fragile micro- and nanostructures that are susceptible to abrasion, UV exposure, and contamination, resulting in gradual loss of water repellency [63,67,68].

Environmental concerns associated with fluorinated compounds further challenge the long-term sustainability of many superhydrophobic systems. Consequently, despite their remarkable water contact angles, superhydrophobic coatings have not achieved the same level of commercial maturity as TiO_2_-based superhydrophilic coatings. The comparison of both strategies is tabulated in Table 6.

### 7.2. Low-Emissivity Versus Thermochromic Coatings

Low-emissivity multilayers and thermochromic coatings represent two distinct approaches to thermal regulation [4,64,65,66,70]. Conventional Low-E coatings utilize highly reflective metallic layers to suppress radiative heat transfer, whereas thermochromic materials such as VO_2_ dynamically modulate infrared transmission in response to temperature changes [65,66].

Low-E coatings have reached high technological maturity because of their excellent optical performance, durability, and compatibility with industrial sputtering processes. In contrast, thermochromic coatings offer adaptive thermal regulation without external energy consumption but suffer from challenges associated with transition temperature, limited optical modulation, fabrication complexity, and cost [65,66].

Although thermochromic coatings have attracted considerable academic interest, their practical deployment remains relatively limited. By contrast, silver-based Low-E multilayers are already extensively employed in commercial glazing applications. Table 7 listed out the comparison between both the coating technologies.

### 7.3. Glass Versus Polymeric Substrates

Glass, polycarbonate (PC), PMMA, and polyethylene each provide distinct advantages and limitations [18,19,20,21,22,23,24,25,26,27].

Glass substrates provide excellent chemical stability and compatibility with high-temperature processing routes. However, brittleness and weight represent important disadvantages. In contrast, polymer substrates offer improved impact resistance and thermal insulation but impose severe constraints on deposition temperature and adhesion.

The choice of substrate frequently determines coating architecture and processing route. Consequently, substrate limitations often become more important than coating chemistry itself in determining practical performance. The comparison among all these substrates is listed in Table 8.

Future advances in greenhouse coatings are therefore expected to rely heavily on low-temperature and flexible processing technologies compatible with polymer substrates.

### 7.4. High-Performance Versus Scalable Manufacturing

Vacuum deposition techniques such as sputtering and ALD produce dense, uniform films with excellent interface quality [35,36,37,38,39,40,41,42,43,44,45]. Solution-based techniques, including spray coating, sol–gel processing, and roll-to-roll manufacturing, provide attractive scalability and lower cost [46,47,48,49,50].

However, a fundamental tradeoff exists between coating quality and manufacturing scalability. Techniques capable of producing highly controlled nanostructures are generally associated with high cost and low throughput, whereas scalable solution-based methods frequently suffer from reduced microstructural control and long-term durability.

This inverse relationship between performance and scalability represents one of the central challenges in translating laboratory coatings into industrial technologies; these are listed in Table 9.

### 7.5. Benchmarking of Current Technologies

Because individual technologies optimize different performance parameters, no single coating architecture currently satisfies all requirements simultaneously. Instead, practical deployment requires balancing optical transmission, thermal regulation, self-cleaning capability, durability, and manufacturing cost. To provide a more quantitative basis for cross-technology comparison, representative optical, thermal, and surface-performance parameters are summarized in Table 10. Where sufficiently comparable data are available, visible-light transmittance, PAR transmission, NIR/solar modulation, SHGC, thermal emissivity, and water contact angle are reported. However, these parameters depend strongly on coating composition, thickness, microstructure, substrate, multilayer architecture, and measurement conditions. Therefore, the values are presented as representative literature values or ranges rather than universal characteristics of each coating class. Parameters for which directly comparable quantitative data are unavailable are indicated as NR.

## 8. Emerging Trends and Future Directions

The field is currently at an important stage in which advances in materials design, fabrication, nanotechnology, and modeling are converging to enable multifunctional glazing systems [127]. Even though today’s glazing coatings can already provide numerous improvements in spectral selectivity and surface engineering, problems still arise with respect to cost-effectiveness, stability, scalability, and multifunctionality, driving the field toward novel directions of development [127,128]. Below are some of the trends that will define the direction of future progress.

### 8.1. Bioinspired and Hierarchical Nanostructures

Nature provides very effective strategies for dealing with light manipulation, wetting behavior, and mechanical robustness that cannot be replicated using conventional engineering methods [129]. The compound eyes of moths consist of sub-wavelength structures called “moth eyes,” which are capable of producing broadband anti-reflective properties (reflectivity less than 1% at wavelengths of 400–1800 nm). On the other hand, the wings of cicadas consist of structurally colored regions along with superhydrophobic regions due to highly ordered nanopillar arrays [129,130]. These hierarchically structured materials with bi-level roughness (10–100 nm + 0.5–2 μm) have been shown to simultaneously enhance light harvesting, self-cleaning, and structural color effects for photosynthetic communication, which could inspire a new generation of glazing coatings [130].

The recent developments in self-assembling block copolymers, nanoimprint lithography, and glancing-angle deposition have made it possible to manufacture these hierarchical structures on glass and polymer surfaces [131]. In terms of bioinspired coatings for greenhouses, there is a potential to achieve exceptional PAR diffusion (>90%) and high transparency (>85%) while retaining superhydrophilic or superhydrophobic properties, which resist wear and tear [131,132]. The unique structural coloring feature of these materials can also offer visual indicators to tune crop-specific light spectrum requirements without any additional LED-based lighting sources [132]. Technologies revolutionizing greenhouse glazing are illustrated in Table 11 through Technology Readiness Levels, with bioinspired nanostructures demonstrating TRL 4–6 for 15% increased PAR diffusivity and ST-PV technologies operating at TRL 6–7 for multifunctional applications in food-energy co-production.

It is important to recognize that the reported Technology Readiness Level (TRL), commercialization potential, and expected deployment timelines of greenhouse coating technologies can vary widely. These differences arise from factors such as coating composition, fabrication techniques, film thickness, compatibility with different substrates, scalability of production, and long-term environmental stability. As a result, the values presented in this review should be considered as approximate indications based on the current body of literature, rather than definitive or universally applicable benchmarks.

### 8.2. Multifunctional and Adaptive Systems (Smart + PV Integration, Dual/Tri-Functional Coatings)

The combination of optical control, energy generation, and environmental interaction within a single-layer system is among the most challenging areas of development [133]. Using semi-transparent perovskite or organic PVs (ST-PV) in glazing materials would provide electricity production of 10–50 W/m^2^ to power greenhouse auxiliary systems using 70–80% of PAR radiation, thus creating food-energy dual-purpose structures [107,133]. Another promising area of research involves luminescent solar collectors (LSCs) that are embedded in polymer-coating materials which would transform UV/blue excess light into red crop-useful radiation [107].

The “smart” nature of the functionality involves taking multifunctionality to the next level by involving responsiveness [119]. Thermochromic materials based on salt hydrates, gels or hybrid perovskites have the potential for broader hysteresis and fast switching compared to VO_2_, while electrochromic oxides, such as WO_3_ and NiO, allow the active modulation of PAR via an applied voltage [119,138]. The emergence of coatings that are tri-functional by being spectral selective + self-cleaning + photovoltaic (or NIR controlling + dewing prevention + energy storage) is expected from high-throughput deposition techniques [138].

### 8.3. Sustainable Materials, Recyclability, and Green Processing

Increasingly, environmental impacts and circular-economy principles are becoming important considerations in the development of functional glazing coatings [99,134,135]. Sustainable coating design should consider not only operational benefits such as reduced heating and cooling demand or improved greenhouse productivity but also the environmental burdens associated with raw-material production, precursor synthesis, solvent use, deposition, post-treatment, maintenance, and end-of-life management. Water-based or low-VOC sol–gel formulations, bio-based precursors, and lower-energy deposition approaches can reduce some of these burdens, while atmospheric-pressure plasma and other solvent-minimized processes may offer opportunities for lower-resource coating fabrication [134,135,139,140]. However, the environmental advantage of a coating cannot be assumed solely from its processing route because energy consumption, precursor chemistry, coating lifetime, and performance gains must be considered together.

Life-cycle assessment (LCA) provides an appropriate framework for evaluating these trade-offs. An anticipatory LCA of a sol-gel-derived anti-reflective coating for greenhouse glass demonstrated that the environmental performance of a coating depends on the complete coating–glazing system and on factors such as production scale, manufacturing location, and assumed service life [99]. Thus, a coating with improved optical transmission is not automatically environmentally preferable if its production requires substantially greater energy or chemical inputs. Conversely, a coating may provide a favorable environmental balance when its additional manufacturing impacts are compensated by improved light utilization, reduced operational energy demand, increased crop productivity, and extended glazing lifetime. Future LCA studies should therefore use consistent functional units and system boundaries and consider both environmental burdens and crop/energy benefits over the expected service life.

Fluorinated surface chemistries require particular environmental consideration. Fluorinated compounds are widely used to obtain low surface energy, water repellency, and chemical resistance in superhydrophobic coatings; however, persistent fluorinated substances, particularly PFAS-type chemistries, raise concerns because of their chemical persistence and potential environmental release. Therefore, the high water-repellency and self-cleaning performance of fluorinated coatings should be evaluated together with their persistence, potential release during manufacture and service, and end-of-life management. These concerns strengthen the motivation for developing fluorine-free hydrophobic and self-cleaning alternatives based on inorganic oxides, silica-based architectures, or other non-fluorinated surface chemistries [135,136].

Recyclability and end-of-life management are particularly challenging for multifunctional multilayer glazing. Conventional glass has established recycling pathways, whereas strongly adhered multilayer coatings containing metals, oxides, polymers, or organic–inorganic components can complicate separation and recovery. Coating removal, selective delamination, reusable substrates, and designs that facilitate material separation could improve the recyclability of coated glazing [139,141]. Accordingly, future multifunctional coatings should be designed not only for high initial optical and thermal performance but also for long service life, repairability, selective removal, material recovery, and compatibility with established glass-recycling streams.

Overall, sustainable greenhouse glazing should be evaluated using a life-cycle perspective that simultaneously considers embodied environmental impacts, processing energy, chemical safety, coating durability, operational energy savings, crop productivity, and end-of-life recovery. The most sustainable coating is therefore not necessarily the coating with the highest initial functional performance, but the one that achieves the required crop-specific optical and thermal functions with low environmental burden, long service life, minimal hazardous chemical use, and a realistic pathway for recycling or recovery.

### 8.4. Data-Driven and AI-Assisted Design of Coating Architectures

Machine learning and generative design technologies have enabled a data-driven approach to coatings research through the acceleration of the old trial-and-error process [137]. Databases consisting of large amounts of data generated using high-throughput libraries formed by multilayer gradient sputtering or inkjet printing precursor arrays provide the training set needed to train neural networks for predicting optimal multilayers for any PAR/NIR/UV target or to train GANs for proposing entirely new hierarchical structures [137,142]. Neural networks trained on such data sets can identify the best multi-layer configurations for any PAR/NIR/UV target, whereas generative adversarial networks generate entirely new hierarchical nanostructures [142].

Computer models that incorporate physics for the propagation of electromagnetic waves, photocatalytic reactions, and thermal/mechanical stresses allow virtual testing of coating structures before their creation [137]. Subsequent optimization via Bayesian methods can help achieve globally optimal performance through experimentation [137]. In the context of greenhouses, such methodologies can allow the generation of “on-demand” coatings that match the particular requirements of different plant species, climatic conditions, and structural configurations while offering digital twinning that allows predicting degradation and planning maintenance [137,143]. Integration of IoC sensors within the layers of functional coatings will bring about adaptive optimization [143].

This confluence of trends places advanced coatings at the core of the future of sustainable agriculture, where the glazing system works to increase crop yields while producing power, conserving resources, and ensuring environmental resilience.

## 9. Conclusions

In this review, the state of the art of optical and self-cleaning coatings for transparent substrates applied in greenhouses has been considered with an emphasis on the interrelation of the chemical composition and morphology with functionality. It has been shown that substantial advances have already been achieved in terms of producing coatings that will increase transparency and control thermal effects while maintaining the cleanliness of the substrate. Dielectric films, photocatalytic oxide coatings, low-emissivity layers, and thermochromic coatings show a lot of promise when used for applications in greenhouses. Nevertheless, there are certain aspects that need further consideration; specifically, durability under the influence of various factors such as UV rays, humidity, and mechanical and chemical abrasion is one of them. Further, several coatings that have been optimized for architectural use would need to undergo additional improvements in order to be suitable for use in greenhouse systems due to their more stringent PAR transmission requirements, as well as their need for environmental compatibility. The future of research and development in this field hinges on the synergy between solid material design principles and effective manufacturing techniques. Methods involving nanostructured surfaces, bioinspired surfaces, hybrid coatings, and data-enabled optimization could provide potential directions for development. Meanwhile, there is a need for greater attention to be devoted to realistic durability studies and life-cycle assessments. Overall, this review highlights that the development of multifunctional greenhouse glazing coatings requires an integrated consideration of materials chemistry, processing, microstructure, functional properties, and environmental durability rather than optimization of a single coating function.

## Figures and Tables

**Table 1:** Representative substrates and compatible functional coatings for greenhouse glazing.

Substrate	Typical Thickness/Form	Key Intrinsic Properties (Uncoated)	Common Coating Types	Main Coating Functions	Representative Refs
Soda-lime glass	3–6 mm rigid panels	High VIS/PAR transmittance, poor insulation, brittle	Dielectric/metal–dielectric stacks; TiO_2_, ZnO; nanostructured silica	Solar control, UV blocking, self-cleaning, AR	[4,7,9,17,21,23,24]
Polycarbonate	Single or multi-wall sheets	Good impact, moderate VIS, better insulation	Sol–gel hard-coats; UV-absorbing and AR layers	Scratch resistance, UV protection, light diffusion/AR	[6,18,22,25,26,27]
PMMA	Solid rigid panels	Very high VIS, good UV stability	AR, anti-fog, self-cleaning sol–gel/hybrid coatings	Surface wetting control, reduced reflection, self-cleaning	[1,22,25,26,27]
PE film (LD/LLDPE)	Thin flexible films	High initial PAR, low insulation, limited lifetime	Thin oxide/hybrid layers; surface active additives	UV stabilization, anti-fog, spectral tuning	[18,21,24]

**Table 2:** Comparison of deposition techniques for greenhouse glazing coatings.

Technique	Typical Substrate Compatibility	Typical Process/Temperature Conditions *	Thickness Control	Uniformity (Large Area)	Cost/Throughput	Key Advantages for Glazing	Key Limitations	References
Magnetron Sputtering	Glass, selected heat-stable polymers	~100–400 °C	Excellent (nm)	High	High/Medium-high	Dense multilayers, precise optical and low-E stacks	Capital intensive; substrate heating	[36,40]
Thermal Evaporation	Mainly Glass, selected heat-stable substrates	~200–600 °C	Moderate-high	Moderate	Medium/Medium	Simple metallic and oxide films	Limited step coverage, Line of sight deposition	[35]
Reactive CVD	Mainly Glass, selected polymers with low-temperature variants	~300–600 °C for conventional processes	Moderate-high	Good	Medium/High	Large-area FTO, pyrolytic coatings	Process-specific precursor and temperature requirements	[37,43]
ALD	Glass and temperature-compatible polymers	~100–300 °C process dependent	Excellent (1Å-scale)	Excellent	High/Low	Conformal ultrathin and barrier layers; precise interface control	Slow deposition and high process cost	[44,45]
Sol–gel Dip/Spin	Glass, polymers	<200 °C process dependent	Moderate	Good	Low-Medium/High	Low-temperature processing, composition flexibility, self-cleaning and hybrid coatings	Shrinkage/cracking, porosity control	[22,46]
Spray Coating	Glass and polymers; potentially large-area substrates	Ambient-150 °C	Moderate	Good when process is optimized	Low/High	Simple, scalable deposition of TiO_2_ and other functional coatings	Spray uniformity and overspray control	[48,49]
Roll-to-roll/Slot-die	Flexible Polymer films	Ambient-120 °C	Good	Excellent for continuous processing	Low-medium/High	Scalable coating of flexible greenhouse films	Web handling, drying, and defect control	[38,48]

* Processing temperature is representative and process-dependent, rather than a universal temperature range.

**Table 3:** Structure–property correlations for key greenhouse coatings.

Coating Type	Microstructure	Optical (*n*, Eg)	Wetting (θ)	Hardness (GPa)	Emissivity (ε)	Greenhouse Benefit	References
TiO_2_ Photocatalytic	Nanocrystalline/Porous, depending on deposition treatment	*n* ≈ 2.5, E_g_ ≈ 3.2 eV	Superhydrophilic, θ < 5° after activation	~1–5 **^†^** GPa	N/A	Photocatalytic self-cleaning, UV management	[58,63]
SiO_2_/TiO_2_ Multilayer	Dense amorphous/oxide multilayer	*n* ≈ 1.46/2.5	Hydrophilic	Process dependent	0.7	Anti-reflection, spectral control	[56,61]
Ag Low-E Stack	Metal/dielectric multilayer with protected Ag	Metallic/dielectric optical response	N/A	Ag ≈ 1–2 **^‡^** Process dependent	<0.05	Low-E thermal control, Winter heat retention	[4,64]
VO_2_ Thermochromic	Polycrystalline film with grain boundaries	Thermochromic IR modulation; E_g_ ≈ 0.7 eV in the relevant state	Hydrophilic	Process dependent	Variable with thermochromic state	Adaptive solar/thermal control	[65,66]
SiO_2_ Superhydrophobic	Hierarchical nano-rough structure	*n* ≈ 1.4–1.5	θ > 150°	≈2–5 Process dependent	0.9	Water shedding/self-cleaning	[67,68]

**Note:** Reported hardness values are representative literature values for specific coating compositions, microstructures, deposition methods, and testing conditions and should not be interpreted as universal values for the corresponding coating class. **†** Representative range for sol-gel-derived TiO_2_ films. **‡** Values depend on layer architecture, thickness, substrate, and measurement conditions. N/A = not applicable or not a principal functional parameter.

**Table 4:** Standardized durability tests for greenhouse coatings.

Test Method	Stressors Simulated	Duration/Cycles	Key Metrics	Greenhouse Relevance	Pass Criteria	References
QUV (ASTM G154)	UV (340 nm) + humidity	2000–4000 h	ΔE* < 5, haze < 5%, θ stable	Solar UV + dew	Transmittance loss < 3%	[94]
Thermal Cycle (EN 1279)	−20/+60 °C	1000 cycles	Tape test pass, Lc > 15 N	Diurnal swings	No blisters/delamination	[95]
Taber Abrasion (D4060)	Mechanical wear	1000 cycles	Haze increase < 5%	Cleaning/sand	PAR loss < 2%	[95]
Salt Fog (B117)	Corrosive atmosphere	1000 h	Visual corrosion, adhesion	Coastal farms	No pitting	[96]
Xenon Arc (ISO 11341)	Full solar spectrum	3000 kJ/m^2^	Combined metrics	Outdoor equivalent	10-year prediction	[97]

**Table 5:** Greenhouse-specific requirements versus conventional glazing requirements.

Parameter	Greenhouse Glazing	Architectural Glazing	Solar/PV Glazing
PAR transmission	Very high priority	Low/moderate	Not primary
NIR control	Crop/thermal balance	Solar heat gain	Device efficiency
UV management	Crop-specific	Usually blocking	Material stability
Self-cleaning	Important	Desirable	Important
Anti-fog	Critical	Less critical	Usually not critical
Agrochemical resistance	Critical	Not relevant	Not relevant
Durability	Agricultural environment	Building environment	Outdoor environment

**Table 6:** Comparison of self-cleaning strategies.

Property	Superhydrophilic (TiO_2_)	Superhydrophobic
Mechanism	Water sheeting	Droplet rolling
Contact angle	<5°	>150°
Photocatalytic activity	Yes	No
Mechanical durability	High	Moderate–Low
UV stability	High	Moderate
Environmental concerns	Low	PFAS concerns
Commercial maturity	High	Medium
Major limitation	UV dependence	Mechanical fragility

PFAS (per- and polyfluoroalkyl substances).

**Table 7:** Comparison of thermal management coatings.

Property	Low-E Coatings	Thermochromic Coatings
Thermal regulation	Passive	Adaptive
Emissivity	<0.05	Variable
Dynamic switching	No	Yes
Manufacturing complexity	Moderate	High
Cost	Moderate	High
Commercial maturity	High	Low
Main limitation	Static behavior	Transition temperature

**Table 8:** Substrate comparison.

Property	Glass	PC	PMMA	PE
Transparency	Excellent	Good	Excellent	Good
Impact resistance	Low	High	Moderate	High
Thermal stability	Excellent	Moderate	Moderate	Poor
Coating compatibility	Excellent	Moderate	Moderate	Limited
Lifetime	High	Moderate	Moderate	Low

**Table 9:** Performance–scalability trade-offs of representative coating deposition routes.

Deposition Route	Film Quality	Cost	Throughput	Scalability	Suitability for Greenhouse Deployment
ALD	Excellent	High	Low	Moderate	High for ultrathin/barrier layers; limited by throughput
Magnetron Sputtering	Excellent	High	Moderate-high	High	High for multilayer optical and low-E coatings
CVD	High	Moderate	High	High	High for large-area oxide coatings where substrate temperature is compatible
Sol–gel	Moderate-high	Low	High	High	High for low-temperature, compositionally flexible coatings
Spray coating	Moderate	Low	Very High	Excellent	High for large-area retrofit and solution-based coatings
Roll-to-roll/slot-die	Moderate-high	Low-moderate	Very High	Excellent	High for flexible polymer greenhouse films

**Table 10:** Quantitative and qualitative benchmarking of representative multifunctional coating technologies for greenhouse glazing.

Coating Technology	Tvis (%)	PAR Transmission (%)	NIR/Solar Control	SHGC	Thermal Emissivity, ε	Water Contact Angle (°)
TiO_2_ photocatalytic	86.95–88.89 *	NR	UV blocking; NIR control architecture-dependent	NR	NR	14.89° after 2 h UV
TiO_2_–SiO_2_ multifunctional sol–gel	>93 *	NR	Primarily anti-reflective; spectral control architecture-dependent	NR	NR	≈1° after UV
Superhydrophobic nanostructured	NR †	NR	Generally limited unless combined with optical-control layers	NR	NR	>150°
Ag-based low-E multilayer	80 ‡	NR	Strong IR/NIR reflection	0.54 ‡	0.048 ‡	NR
VO_2_ thermochromic	44.2–76 §	NR	ΔTsol = 5.2–17.7%; ΔTIR up to 13.5%	NR	variable	NR
Hybrid/multifunctional sol–gel	System-dependent	NR	Architecture-dependent	NR	System-dependent	System-dependent

Note: Tvis = visible-light transmittance; PAR = photosynthetically active radiation (400–700 nm); NIR = near-infrared; SHGC = solar heat gain coefficient; ε = thermal emissivity; NR = not reliably reported or not directly comparable in the cited study. Values are representative literature results for specific coating/substrate configurations and should not be interpreted as universal properties of the corresponding coating class. Optical and thermal performance depends on composition, coating thickness, microstructure, and multilayer architecture, substrate, and measurement conditions. Although PAR transmission is a critical greenhouse-specific parameter, directly reported PAR values were not consistently available for the coating systems considered; therefore, PAR transmission was not inferred from Tvis and is reported as NR where direct measurements were unavailable. * Representative TiO_2_ coating on glass; reported Tvis = 86.95–88.89% and water contact angle decreased to 14.89° after UV irradiation [122]. For a TiO_2_–SiO_2_ sol–gel coating, average transmittance > 93% over 300–1000 nm and water contact angle ≈ 1° after UV irradiation were reported [123]. † The superhydrophobic category is characterized primarily by surface wettability; representative studies report water contact angles above 150°, while optical transmittance varies with nanoparticle composition and substrate [70]. ‡ For a sputtered AlN–Ag low-E coating, the reported Tvis = 80%, SHGC = 0.54, and thermal emissivity ε = 0.048 [124]. § Representative VO_2_ systems demonstrate substantial dependence on film architecture and thickness. A VO_2_/SiO_2_ coating reported Tlum = 44.2–52.5% with ΔTsol = 11.6–17.7%, while a Sn–W co-doped VO_2_ film reported Tlum = 76%, ΔTsol ≈ 5.2%, and ΔTIR = 13.5% [125,126].

**Table 11:** Emerging coating technologies mapped by Technology Readiness Level (TRL) and expected greenhouse impact.

Technology	Estimated TRL	Key Performance	Greenhouse Impact	Representative Commercialization Timeline	Refs
Bioinspired hierarchical AR	4–6	T > 90%, θ > 150°	+15% PAR uniformity	3–5 years	[130,132]
ST-PV glazing	6–7	20 W/m^2^ + 75% PAR	Energy autonomy	2–4 years	[107,133]
4th-gen thermochromics	5–6	ΔT_sol > 15%, Tc < 30 °C	−25% cooling	4–6 years	[119]
Water-based hybrids	7–8	VOC < 10 g/L, H > 5 GPa	Sustainable scale-up	1–3 years	[134,135]
ML-optimized multilayers	3–5	Custom PAR/NIR	Crop-specific	5–7 years	[136,137]

## Data Availability

No new data were created or analyzed in this study. Data sharing is not applicable to this article.
